# Societal attitudes and structural barriers in coaching para-athletes: A mixed-methods systematic review

**DOI:** 10.1371/journal.pone.0326585

**Published:** 2025-06-25

**Authors:** Junyan Liu, Hongjun Yu, Waifong Catherine Cheung, Adam Bleakney, Yih-Kuen Jan

**Affiliations:** 1 Department of Physical Education, Tsinghua University, Beijing, China; 2 Department of Health and Kinesiology, University of Illinois at Urbana-Champaign, Urbana, Illinois, United States of America; 3 Doctor of Physical Therapy Program, Northern Illinois University, DeKalb, Illinois, United States of America; 4 Disability Resources and Educational Services, University of Illinois at Urbana-Champaign, Champaign, Illinois, United States of America; Sheffield Hallam University, UNITED KINGDOM OF GREAT BRITAIN AND NORTHERN IRELAND

## Abstract

Coaching athletes with disabilities is essential for promoting inclusivity and equity in sports. However, societal attitudes (e.g., stigmatization and ableism) and structural barriers (e.g., insufficient funding and limited access to adaptive equipment) persist as significant obstacles to their full participation and optimal performance. This systematic review addresses a critical gap in the existing literature by examining the complex interaction between societal and structural factors and their impact on coaching practice for athletes with disabilities. A mixed-methods approach was employed to synthesize data from 26 studies sourced from PubMed, Web of Science, SPORTDiscus, and Google Scholar. By integrating both qualitative and quantitative evidence, this review explores how societal biases and resource limitations converge to create systemic challenges that hinder coaching effectiveness. This review indicates that societal perceptions, including ableism and underestimation of athletic potential, in conjunction with structural barriers, such as inadequate facilities and funding, significantly undermine the effectiveness of coaching practices. Moreover, the findings emphasize the urgent need for comprehensive reforms in coach education and policy implementation with a particular focus on improving accessibility and ensuring the equitable distribution of resources. Ultimately, the evidence suggests that addressing these interconnected barriers is essential for fostering a more inclusive and equitable coaching environment for athletes with disabilities.

## Background

Coaching athletes with disabilities is essential for promoting inclusivity and equity in sports by significantly enhancing the participation and performance of these athletes across both competitive and recreational levels [1,2]. Key variables in effective coaching include not only technical proficiency but also a deep understanding of the physiological, psychological, and social needs of athletes with disabilities. According to a systematic review, professional, interpersonal, and intrapersonal competencies are critical in shaping the relationship between coaches and athletes with disabilities [2]. These domains encompass essential coaching skills, such as sport-specific expertise, adaptive communication strategies, and ongoing self-reflection, all of which are necessary to address the complex challenges faced by athletes with disabilities.

While prior systematic reviews have provided valuable insights into the competencies of coaches, it largely focuses on internal factors and individual relationships, leaving external influences underexplored [3]. The primary issue addressed in this review is the lack of understanding of how societal attitudes and structural barriers intersect with coaching practices in the context of athletes with disabilities. This systematic review aims to examine these external factors, focusing on how they affect the coaching dynamics and athlete development in disability sports. For example, societal attitudes and structural barriers are key external factors that remain insufficiently addressed in existing research [4]. These external influences are particularly significant because they create systemic challenges that affect the effectiveness of coaching strategies. Understanding these barriers is critical to advancing coaching practices and ensuring equitable opportunities for athletes with disabilities [2].

Societal attitudes refer to the collective perceptions, beliefs, and biases held by the public and sports stakeholders toward athletes with disabilities. These attitudes often manifest in the form of stigmatization, ableism, and underestimation of athletes’ capabilities, which can hinder athletes’ access to equitable opportunities. A particularly significant challenge lies in addressing these entrenched biases, as they disproportionately affect marginalized groups, such as female athletes or athletes from economically disadvantaged backgrounds, who may face compounded barriers due to intersecting forms of discrimination. Structural barriers, on the other hand, encompass tangible and systemic challenges such as inadequate sports facilities, limited financial resources, and insufficient institutional support for adaptive training environments [5]. While existing literature has begun to document these challenges [6], there remains a significant gap in understanding how these factors intersect with coaching practices. For example, 65% of coaches report observing societal biases that influence team selection, and these biases contribute to reduced opportunities for athletes with disabilities at the elite level, as well as adverse mental health outcomes [7].

In addition to societal biases, structural barriers exacerbate the challenges faced by both coaches and athletes. Studies have shown that 40% of training venues lack essential adaptive equipment, severely restricting athletes’ ability to train effectively. Such deficits not only hinder athletic development but also perpetuate societal stereotypes about the perceived limitations of athletes with disabilities. These challenges are further compounded by systemic inequities in policy implementation, where resource allocation often prioritizes able-bodied sports, leaving disabled sports underfunded and inadequately supported. These structural challenges have been further compounded by the COVID-19 pandemic, which disrupted traditional coaching practices and disproportionately affected athletes with disabilities [8,9]. During the pandemic, over 60% of athletes with disabilities reported reduced access to adequate coaching support, highlighting the vulnerability of this group in the face of systemic inadequacies [10]. Despite growing awareness of disability sports, these findings illustrate persistent barriers to achieving true inclusivity in sports environments.

While previous reviews have extensively documented the impact of these barriers on athletes’ performance [11], less attention has been given to how coaches navigate these external challenges. Coaches, as key figures in the sports ecosystem, play a critical role in mitigating the effects of societal attitudes and structural impediments. According to Wareham et al., coaches act as intermediaries, translating societal and institutional support (or lack thereof) into effective coaching practices that can either facilitate or hinder athlete development. Existing reviews have focused primarily on the individual competencies required for coaching athletes with disabilities but have not fully explored the broader societal and structural contexts that shape these practices [2]. This creates a critical gap in the literature regarding how coaches adapt to external pressures while maintaining an inclusive and supportive training environment [12].

Building upon these foundational insights, this systematic review employs a mixed-methods approach to address the gap in the literature by examining the interplay between societal attitudes and structural barriers in disability sports coaching [13]. By integrating both qualitative and quantitative data, the review explores how societal attitudes, such as stigmatization and ableism, affect coaching dynamics and athlete development, and how systemic challenges posed by structural barriers, such as inadequate facilities and resources, influence coaching practices. Through synthesizing current literature and proposing practical, evidence-based strategies, this paper contributes to the growing discourse on disabled sports by expanding the focus from internal coaching competencies to external influences, offering both theoretical and practical guidance for fostering greater inclusion and promoting performance equity in sports environments.

## Method

This review employs an integrative mixed-methods review approach combined with selected elements of PRISMA (Preferred Reporting Items for Systematic Reviews and Meta-Analyses) to synthesize both qualitative and quantitative evidence systematically [14,15]. The integrative review methodology was chosen to accommodate the inclusion of diverse evidence types, including empirical, theoretical, and policy-oriented studies, providing a holistic understanding of societal attitudes and structural barriers in coaching athletes with disabilities [14]. To ensure transparency and rigor, key elements from PRISMA, such as a structured literature search strategy, detailed documentation of study selection, and clear inclusion and exclusion criteria, were incorporated [15].

### Problem identification and review objectives

This integrative mixed-methods review synthesizes qualitative and quantitative evidence to explore how societal attitudes and structural barriers influence coaching practices for athletes with disabilities. By examining themes in societal attitudes and analyzing structural barriers, the review provides a comprehensive understanding of their combined impact on inclusive coaching practices. The objectives are to identify key patterns in the existing literature, address gaps in knowledge, and offer insights to inform future research and policy. This approach integrates diverse evidence sources to present a holistic perspective on the challenges and opportunities in coaching athletes with disabilities.

### Search strategy

This review employed a detailed search strategy aligned with the Preferred Reporting Items for Systematic Reviews and Meta-Analyses (PRISMA) guidelines to identify studies examining societal attitudes and structural barriers in coaching athletes with disabilities. A systematic four-step process including identification, screening, eligibility, and inclusion was followed to ensure the rigorous selection of relevant qualitative and quantitative studies [15]. In addition, a search was conducted on the National Institute for Health and Care Research’s International Prospective Register of Systematic Reviews (PROSPERO) to confirm that no ongoing reviews on this topic existed. This review was registered with PROSPERO under registration number CRD42024622486.

### Databases and search terms

The following electronic bibliographic databases were comprehensively searched from the earliest record to August 2024 to identify relevant studies: PubMed, Web of Science, and SPORTDiscus. Additional searches were performed in Google Scholar to capture supplementary studies and grey literature. The search terms were developed based on key concepts related to societal attitudes, structural barriers, and coaching athletes with disabilities. Boolean operators (AND, OR) were employed in the bibliographic databases to combine search terms systematically and ensure a thorough search. In Google Scholar, a more flexible, free-text approach was adopted, incorporating various keywords and phrases to identify relevant resources. The specific search strategy utilized across databases and search engines included the following terms and combinations: (disabled athletes[Title/Abstract] OR para-athletes[Title/Abstract] OR disabilities[Title/Abstract] OR Paralympic Athletes[Title/Abstract] OR Wheelchair Athletes[Title/Abstract] OR Intellectual Impairments[Title/Abstract] OR Mobility Impairments[Title/Abstract] OR Special Athletes[Title/Abstract] OR Sportspeople with Disabilities[Title/Abstract] OR Parasport[Title/Abstract]) AND (societal attitudes[Title/Abstract] OR social barriers[Title/Abstract] OR ableism[Title/Abstract] OR discrimination[Title/Abstract] OR stigma[Title/Abstract] OR stigmatization[Title/Abstract] OR societal perceptions[Title/Abstract] OR inclusion[Title/Abstract] OR structural barriers[Title/Abstract] OR accessibility[Title/Abstract] OR funding limitations[Title/Abstract] OR lack of funding[Title/Abstract] OR underfunded[Title/Abstract] OR facilities[Title/Abstract] OR infrastructure[Title/Abstract] OR coach[Title/Abstract] OR retaliation[Title/Abstract] OR sociopolitical factors[Title/Abstract]).

### Inclusion and exclusion criteria

The inclusion and exclusion criteria for this systematic review were structured around the PICO (Population, Intervention, Comparison, Outcome) framework to enhance methodological transparency and reproducibility [16].

Population (P): The target population included athletes with disabilities, coaches, and support staff, as all these groups contribute to shaping the coaching experience in disabled sports.

Intervention (I): Studies examining the influence of societal attitudes, structural barriers, or specific coaching practices on the coaching of athletes with disabilities were included. This encompasses factors such as stigmatization, lack of funding, inadequate facilities, or adaptive coaching strategies.

Comparison (C): Studies with or without comparison groups (e.g., disabled versus non-disabled athletes, coached versus un-coached athletes) were eligible, provided they addressed the intersection of societal attitudes, structural barriers, and coaching practices.

Outcomes (O): Relevant outcomes included, but were not limited to, changes in coaching practices, accessibility, athlete performance, participation rates, and psychosocial impacts on athletes or coaches.

To ensure a rigorous and comprehensive review, the inclusion criteria encompassed studies employing quantitative, qualitative, and case study designs that addressed the interaction between societal attitudes, structural barriers, and coaching practices. Only peer-reviewed journal articles published in English were included to maintain academic rigor. No restrictions were applied to the publication year, allowing the inclusion of both historical and contemporary perspectives on the topic.

The exclusion criteria aimed to maintain the focus and quality of the review. Non-academic sources, such as media reports, blogs, and opinion pieces, were excluded due to their lack of scholarly rigor. Studies that did not directly address the intersection of societal attitudes, structural barriers, and coaching practices were omitted. Additionally, duplicate publications and those with substantial content overlap were removed. Articles lacking accessible full texts or methodological rigor, such as robust research designs or analytical techniques, were excluded to ensure the reliability of the included studies [17].

### Data extraction

Data from the selected studies were systematically extracted using a standardized form (Table 1) tailored to the objectives of this review. The form captured essential study characteristics, including title, authors, publication year, research design, and participant demographics (e.g., types of disabilities, sports involvement). To ensure relevance to the thematic analysis, key findings explicitly related to societal attitudes and structural barriers were also extracted as analytical data. To facilitate a structured analysis, a thematic analysis code table (Table 2) was developed to categorize the extracted findings. This table provided a clear coding system, enabling the systematic identification and grouping of themes such as “Societal Attitudes,” “Structural Barriers,” and “Coaching Barriers” across the studies. Each study’s findings were linked to one or more of these themes, represented by simple code labels, which helped streamline the analysis process and ensure consistency in the synthesis. These findings included direct quotes, summarized results, and any interpretative insights reported in the studies. To ensure accuracy and consistency, two independent reviewers screened titles and abstracts, followed by a full-text review. Disagreements were resolved through discussion or consultation with a third reviewer when necessary. The extracted data were prepared for thematic analysis, forming the foundation for coding, theme development, and synthesis.

**Table 1:** Summary of included articles in the review.

Studies	Country	Subjects			Sports	Disability	Method
		Gender	Age	Occupation			
William R. Falcão et al. (2015)	Canada	Male	Average age of 42.67 years	Head coaches of summer and winter Paralympic sport teams	Includes individual and team sports (e.g., athletics, swimming, etc.)	Athletes with various impairments such as cerebral palsy, spinal-cord injuries, and amputations	Semi-structured interviews and thematic analysis
Y. Wareham et al. (2017)	Australia	9 males, 3 females	Not specified	Coaches of elite athletes with disabilities	Includes swimming, athletics, cycling, canoeing, equestrian sports, wheelchair basketball, and more	Vision impairment, amputation or limb deficiency, spinal cord injury, cerebral palsy	Semi-structured interviews
Y. Wareham et al. (2018)	Australia	Not specified	Not specified	Senior sports administrators	Athletics, swimming, wheelchair basketball, wheelchair rugby, equestrian sports, and more	Focuses on elite athletes with physical disabilities (no further detail on the extent of impairments provided)	Semi-structured interviews
Janna L. Crawford and Monika Stodolska (2008)	Kenya	Both male and female	27–55 years old	Elite athletes with disabilities	Powerlifting, swimming, athletics, wheelchair basketball, and table tennis	Varied disabilities, including polio and amputation	Grounded theory research, in-depth interviews with athletes and administrators
Lara Pomerleau-Fontaine (2021)	Canada	Both male and female	Adults	Elite wheelchair basketball athletes with acquired disabilities	Wheelchair basketball	Acquired impairments (e.g., spinal cord injuries, other trauma)	Semi-structured interviews with thematic analysis
Danielle Alexander, Gordon A. Bloom, and Shaunna Taylor (2019)	Canada	Female	Not specified	Female Paralympic athletes	Not specified but related to Paralympic sports	Various physical disabilities	Semi-structured interviews, thematic analysis
Manuel Rodríguez Macías et al. (2023)	Spain	Female	Mean age 38.57 years	Spanish Paralympic women athletes	Swimming, athletics, judo, goalball, and cycling	Visual, intellectual, or physical disabilities	Qualitative interviews focusing on social, sporting, and psychological factors
Danielle Alexander et al. (2024)	North America and Europe (Canada, Norway, Sweden)	Both male and female	Athletes ranged from under 20 to over 40 years old	Athletes, coaches, and support teams from national Paralympic teams	Not specified but involves national Paralympic teams	Various physical disabilities (e.g., spinal cord injuries, short stature)	Focus groups and interviews using thematic analysis
Andrea Bundonet al. (2022)	Canada	Both male and female	Ages 21–38	Seven Paralympic hopefuls	Para-athletics, para-cycling, para-triathlon, sitting volleyball, and wheelchair basketball.	Specific disabilities include lower limb impairments	Semi-structured interviews and thematic analysis of psychological wellbeing during the COVID-19 pandemic.
Majed M. Alhumaid et al. (2022)	Saudi Arabia	Both male and female	Aged 24–58 years old	Athletes with disabilities	Athletics, shooting, powerlifting, and wheelchair basketball.	Disabilities include poliomyelitis, paraplegia, amputations, motor disabilities, and cerebral palsy	Interpretive phenomenological analysis of interviews with 26 stakeholders including para-athletes, coaches, and administrators
Gallardo de León, Ponciano Nuñez, Calderón Santos (2024)	Guatemala	Both male and female	Ages 18–52	Eleven para-athletes	Goalball and para-athletics	Visual disabilities in goalball athletes, and various other physical impairments in para-athletics	Qualitative case study based on semi-structured interviews, observations, and field notes
Beth Aitchison et al. (2020)	United Kingdom	Not specified	Not specified	British Paralympic swimmers	Swimming	Disabilities include physical, visual, and intellectual impairments (S1-S14 classification system)	Hermeneutic phenomenological study using in-depth semi-structured interviews
Olga Kolotouchkina et al. (2021)	Japan	Not specified	Not specified	Focus on athletes and public media narratives	Various Paralympic sports covered by media communication strategies		Exploratory case study focusing on media narratives and disability representation during the Tokyo 2020 Paralympics
Aglaja Busch et al. (2022)	Germany	40 women, 38 men	29.8 ± 11.4 years	Paralympic athletes	Various (not specified)	Physical and sensory impairments	Longitudinal observation with Patient Health Questionnaire (PHQ-4)
Marte Bentzen et al. (2022)	Sweden	8 men, 5 women	18–65 years	Elite Para athletes	Para alpine skiing, Para cross country skiing, Wheelchair curling	Physical and visual impairments	Mixed-method, weekly survey using PHQ-4
Damian Haslett et al. (2022)	Ireland	15 men, 13 women	18–49 years	Para athletes	10 sports including Wheelchair Basketball, Para Athletics, Para Swimming	Spinal cord injury, spina bifida, visual impairment, cerebral palsy, amputations	Qualitative interviews focusing on social, sporting, and psychological factors
Emily Anne Rutland et al. (2022)	Ghana, India, Brazil	5 men, 6 women	20–50 years	Para athletes	Para athletics, Para football, Para tennis, Para swimming	Limb deficiency, impaired muscle power	Qualitative focus groups
Janine Coates & P. David Howe et al. (2023)	United Kingdom	Not specified	Children under 25	Parents of young elite para-athletes	Various parasports, including athletics, swimming, and wheelchair sports	Physical disabilities (e.g., wheelchair users, short stature)	Qualitative interviews with 11 parents
Ian Brittain (2024)	United Kingdom	Not specified	Adult	Paralympic athletes	Great Britain Paralympic track and field squad athletes	Includes athletes with visual, physical, and cerebral palsy impairments	Qualitative life-history interviews with 12 athletes
Joanna Sobiecka et al. (2019)	Poland	Not specified	32 ± 10.6 years	Paralympic athletes and coaches	Various Paralympic sports	Locomotor and visual impairments	Survey-based retrospective study
Lisa S. Olive et al. (2022)	Australia	Not specified	Mean 29.5 years for para athletes	Elite para- and non-para athletes	Various para sports	Physical and visual impairments, with some intellectual impairments	Cross-sectional anonymous online survey
Yvette Wareham et al. (2018)	Australia	Both male and female athletes, including three female and nine male coache	Not specified	Coaches of elite athletes with disabilities.Elite athletes with disabilities coached by 12 coaches across various sports.	Swimming, athletics, cycling, canoeing, triathlon, equestrian sport, and wheelchair basketball.	Includes athletes with vision impairment, amputation, spinal cord injury, and cerebral palsy.	Semi-structured interviews and thematic analysis.
Jacqueline Martins Patatas et al. (2020)	Brazil	Not specified	The stakeholders were between 28 and 65 years old	Coaches, sport managers, high-performance directors, and classifiers	Para-athletics, para-swimming, para-powerlifting, wheelchair basketball, and goalball	Various types of disabilities, including congenital and acquired impairments.	Semi-structured interviews with 32 stakeholders.
Melinda A. Maikaet (2014)	Canada	Not specified	Not specified	Paralympic athletes	Various sports covered during the London 2012 Paralympic Games.	Various types of disabilities.	Media content analysis.
Darda J. Sales et al. (2022)	Canada	Not specified.	Not specified.	Para-swimmers and their families	Para-swimming.	Physical impairments related to swimming	Interviews and analysis comparing to the Long-Term Athlete Development model
Michelle Grenier et al. (2022)	Latvia	Both male and female athletes.	Ages ranging from 10 to 22	Wheelchair tennis athletes	Wheelchair tennis athletes	Subjects had various physical disabilities, including spina bifida, cerebral palsy, and post-stroke conditions	Qualitative study using thematic analysis, involving field notes, focus group interviews, and semi-structured individual interviews with athletes, caregivers, and coaches.

**Table 2:** Thematic analysis code table.

Studies	Result	Themes	Code
William R. Falcão et al. (2015)	Lack of specialized training and support for coaches working with athletes with disabilities; Inadequate facilities, funding, transportation, and difficulties in integrating disabled athletes into non-disabled teams.	**Coaching Barriers**	CB
Y. Wareham et al. (2017)	Societal stigma against coaching athletes with disabilities discourages coaches due to reputation concerns; Inadequate facilities, funding, transportation, and difficulties in integrating disabled athletes into non-disabled teams; Lack of disability-specific knowledge and concerns about coaching athletes with impairments.	**Societal Attitudes;** **Structural Barriers;** **Coaching Barriers**	SA; SB; CB
Y. Wareham et al.(2018)	Coaching athletes with disabilities is stigmatized as less elite than non-disabled sports; Barriers include lack of accessible facilities, transportation, financial resources, government support, and ethnic favoritism; Disability sports face limited funding, equipment shortages, and insufficient media coverage; Recruitment is hindered by lack of disability knowledge, but retention improves once coaches work with disabled athletes.	**Societal Attitudes;** **Structural Barriers;** **Coaching Barriers**	SA; SB; CB
Janna L. Crawford and Monika Stodolska (2008)	Negative societal attitudes, such as viewing disability as a curse, lead to social exclusion; Barriers include lack of accessible facilities, transportation, financial resources, government support, and ethnic favoritism.; Few qualified coaches in disability sports, with most coaching done by under-resourced volunteers.	**Societal Attitudes;** **Structural Barriers;** **Coaching Barriers**	SA; SB; CB
Lara Pomerleau-Fontaine (2021)	Negative coaching behaviors, like yelling and ignoring gender differences, harm the coach-athlete relationship.	**Coaching Barriers**	CB
Danielle Alexander, Gordon A. Bloom, and Shaunna Taylor (2019)	Female Paralympic athletes experience inappropriate behavior from male coaches, affecting their psychological well-being; Coaching barriers for female Paralympic athletes include body image comments, sexual harassment, and lack of female coaches.	**Societal Attitudes;** **Coaching Barriers**	SA; CB
Manuel Rodríguez Macías et al. (2023)	Financial challenges and lack of media visibility create structural barriers for athletes.	**Structural Barriers**	SB
Danielle Alexander et al. (2024)	Coaches struggle to manage teams with athletes of varying disabilities, requiring adaptable coaching styles.	**Coaching Barriers**	CB
Andrea Bundonet al. (2022)	Paralympic athletes were marginalized during the COVID-19 pandemic, with societal narratives portraying them as more vulnerable, fostering feelings of inferiority; Athletes had limited access to training facilities and fewer competitive opportunities than able-bodied athletes during the pandemic.	**Societal Attitudes;** **Structural Barriers**	SA; SB
Majed M. Alhumaid et al. (2022)	The program challenges negative societal views of disabilities, aiming to change perceptions of athletes with disabilities as less capable, focusing on empowerment and inclusion in Saudi Arabia’s sports scene; Key barriers include limited media coverage of disability sports, inadequate facilities, and insufficient psychological support for athletes; The program highlights the need for more qualified coaches and improved coaching resources for para-sports.	**Societal Attitudes;** **Structural Barriers;** **Coaching Barriers**	SA; SB; CB
Gallardo de León, Ponciano Nuñez, Calderón Santos (2024)	Discrimination and lack of societal understanding of disabilities are significant barriers affecting athletes’ daily lives; A shortage of trained coaches limits athlete development and performance.	**Societal Attitudes;** **Coaching Barriers**	SA; CB
Beth Aitchison et al. (2020)	Barriers such as poor urban infrastructure, inaccessible transportation, and inadequate sports facilities restrict para-sport participation.	**Structural Barriers**	SB
Olga Kolotouchkina et al. (2021)	Reduced access to training facilities during the pandemic impacted athletes’ mental health and training; Insufficient contact with coaches during the pandemic affected athletes’ performance and mental health.	**Structural Barriers;** **Coaching Barriers**	SB; CB
Aglaja Busch et al. (2022)	The Paralympic Games help shift societal attitudes by increasing visibility and breaking down disability stereotypes; The study emphasizes media representation and technology’s role in improving accessibility, rather than focusing on structural barriers in sports.	**Societal Attitudes;** **Structural Barriers**	SA; SB
Marte Bentzen et al. (2022)	Para athletes face challenges like travel difficulties, lack of adapted healthcare, and high costs of impairment-specific equipment.; While coaches were aware of mental health issues, no specific coaching barriers were mentioned.	**Structural Barriers;** **Coaching Barriers**	SB; CB
Damian Haslett et al. (2022)	Para athlete activism combats societal discrimination and ableism, using platforms to challenge societal perceptions of disability; Structural inequalities in Para sports include limited disability representation and scarce resources for less “able-bodied” athletes.	**Societal Attitudes;** **Structural Barriers**	SA; SB
Emily Anne Rutland et al. (2022)	Athletes faced stigma and societal neglect, leading to exclusion and marginalization in both sporting and non-sporting contexts; Financial exploitation, neglect, and systemic discrimination were structural barriers affecting athletes in low-resource countries.; Some coaches were complicit in abuse or neglect, though specific coaching barriers were not deeply explored.	**Societal Attitudes;** **Structural Barriers;** **Coaching Barriers**	SA; SB; CB
Janine Coates & P. David Howe et al. (2023)	Parents raised concerns about societal exclusion in mainstream schools, driving them to seek alternative sporting opportunities for their children; Parents identified a lack of local para-sport opportunities and resources for disabled athletes as major barriers; Parents noted that some coaches lacked the knowledge to properly support their disabled children’s athletic development.	**Societal Attitudes;** **Structural Barriers;** **Coaching Barriers**	SA; SB; CB
Ian Brittain (2024)	Negative societal perceptions, rooted in the medical model, reinforce stereotypes and limit disabled individuals’ involvement in sports and social integration; Lack of access to appropriate sports facilities and opportunities serves as a key structural barrier at all levels.	**Societal Attitudes;** **Structural Barriers**	SA; SB
Joanna Sobiecka et al. (2019)	Polish sports organizations show limited understanding and appreciation for disabled athletes, leading to underrepresentation and weak cooperation; Key barriers include insufficient funding, inadequate infrastructure, limited access to specialized equipment, and poor medical, nutritional, and psychological support.; A shortage of qualified coaches and limited access to disability-specific coaching hinder athlete development.	**Societal Attitudes;** **Structural Barriers;** **Coaching Barriers**	SA; SB; CB
Lisa S. Olive et al. (2022)	Para athletes face discrimination, affecting their mental health and increasing social isolation; Barriers for para athletes include venue access, travel costs, and equipment challenges, negatively impacting mental health.	**Societal Attitudes;** **Structural Barriers**	SA; SB
Yvette Wareham et al. (2018)	Social-cultural barriers and stigma limit participation, causing exclusion in both the community and sports organizations.	**Societal Attitudes**	SA
Jacqueline Martins Patatas et al. (2020)	Stigma and cultural barriers restrict athletes with disabilities from participating in sports; The complex classification system limits opportunities for athletes with severe disabilities.; Coaches lack disability-specific knowledge and training.	**Societal Attitudes;** **Structural Barriers;** **Coaching Barriers**	SA; SB; CB
Melinda A. Maikaet (2014)	Media portrayal of Paralympians as “supercrips” reinforces ‘othering’ and harmful stereotypes.	**Societal Attitudes**	SA
Darda J. Sales et al. (2022)	Social perceptions of disability hinder para-athletes’ integration into mainstream sports. Lack of knowledgeable coaches and adapted training environments, combined with classification issues, restricts competition; Coaches lack expertise in adapting training for para-athletes.	**Societal Attitudes;** **Structural Barriers;** **Coaching Barriers**	SA; SB; CB
Michelle Grenier et al. (2022)	Athletes and families report societal exclusion and negative perceptions, leading to isolation; Inaccessible facilities and support programs, along with societal attitudes, limit participation.	**Societal Attitudes;** **Structural Barriers**	SA; SB

### Quality assessment

The methodological quality of the included studies was assessed using the Mixed Methods Appraisal Tool (MMAT) [18], selected for its ability to comprehensively evaluate qualitative, quantitative, and mixed-methods studies. Mixed methods research is an approach that combines qualitative and quantitative methods to address complex research questions that cannot be fully answered by either method alone [19,20]. The MMAT evaluates key aspects of research design, including clarity of research questions, appropriateness of methodologies, reliability of data collection instruments, transparency in participant selection, and rigor of data analysis, with an emphasis on triangulation, which enhances the validity of results, and completeness, where qualitative data explain quantitative findings, offering a fuller picture [21].

Each study was systematically evaluated using MMAT’s 5-point scale. Studies scoring 5 (*****) demonstrated excellence in research design and rigor, indicating high methodological soundness and minimal bias. Mixed methods research, rooted in pragmatism, allows researchers to select and integrate methods based on the research question, overcoming the limitations of each approach when used independently [22]. Studies scoring 3 (***) or 4 (****) met most MMAT criteria but exhibited some limitations, such as less transparency in participant selection or limited triangulation. Low-scoring studies (1 or 2) were included for comprehensiveness but critically assessed during synthesis to minimize bias.

To evaluate the certainty of the synthesized findings, the GRADE-CERQual (Confidence in the Evidence from Reviews of Qualitative Research) framework was applied [23]. CERQual assesses four key components: methodological limitations, coherence, adequacy of data, and relevance. The MMAT results informed the “methodological limitations” component, while the overall evaluation assigned confidence ratings (high, moderate, low, or very low) to each finding [20]. By integrating qualitative and quantitative methods, mixed methods research allows researchers to gain a more comprehensive and holistic understanding of phenomena, combining both depth and breadth in their findings. By integrating MMAT and CERQual, this study ensured a systematic and transparent approach to assessing the robustness and reliability of the evidence base.

### Data synthesis

A thematic synthesis was conducted following Braun and Clarke’s six-phase framework, which emphasizes systematic and iterative analysis to identify and refine themes [24]. Braun and Clarke’s method is a form of thematic analysis used to identify, analyze and report themes within qualitative data. This process began with familiarization, where data from the 27 included studies were read repeatedly, and initial notes were taken to highlight key concepts. The next step involved generating descriptive and interpretative codes that captured recurring patterns related to societal attitudes and structural barriers. These codes were then organized into categories based on the identified themes, such as “biases,” “stereotypes,” and “access to resources.” The coding process was iterative, involving multiple rounds of analysis where codes were refined and cross-checked for consistency across the data sources. This iterative process ensured that the themes aligned with the research objectives and accurately reflected the data.

Quantitative data were incorporated into the thematic synthesis as contextual evidence to complement qualitative findings. The integration of quantitative data occurred at the coding level, where numerical results were assigned to corresponding qualitative themes. For example, survey percentages illustrating societal biases were incorporated into the theme of “societal stigmas,” while frequencies of reported barriers were aligned with themes such as “structural inequities.” This convergence approach involved interpreting quantitative results descriptively and aligning them with qualitative insights to support overarching themes. By doing so, the quantitative data enriched qualitative synthesis, providing a more comprehensive understanding of the phenomena. The convergence approach is aligned with Braun and Clarke’s method on the theme identification and is useful for the integration of qualitative and quantitative data [24,25].

The analysis identified three major thematic categories: societal attitudes, structural barriers, and their intersectional impact on coaching practices (Fig 2). Within societal attitudes, recurring patterns included biases, stereotypes, and cultural stigmas that shaped coaching environments, often undermining inclusivity and misjudging athletes’ potential. Structural barriers encompassed limited access to facilities, insufficient resources, and inadequate training for coaches on disability-specific needs. These barriers significantly constrained efforts to create inclusive coaching practices.

Through iterative refinement, the analysis revealed a dynamic interplay between societal attitudes and structural barriers. Negative societal perceptions often reinforced structural inequities, contributing to systemic underfunding and inadequate infrastructure. For example, stigmatization led to reduced resource allocation, perpetuating institutional challenges that hindered inclusive coaching practices. The iterative process of coding and theme refinement allowed for clear categorization of these complex relationships, ensuring that the final thematic categories were robust and aligned with the research objectives.

To ensure transparency and rigor, an audit trail was maintained throughout the analysis process, documenting coding decisions, theme development, and integration of quantitative data. This thematic synthesis underscores the critical need for institutional reforms. Addressing both societal attitudes and structural barriers is essential for fostering inclusive coaching practices. Key recommendations include improving accessibility, increasing resources for coaches, and implementing targeted training programs tailored to the unique needs of athletes with disabilities. By integrating quantitative findings (Table 3) into the thematic framework, this synthesis provides a comprehensive and nuanced understanding of the challenges and opportunities for promoting equity in disability sports coaching.

**Table 3:** Summary of quantitative findings from included studies.

Study	Sample Size	Population/Setting	Methodology	Outcome Measures	Key Quantitative Results	Limitations
Aglaja Busch et al.(2022)	78 para-athletes; 78 controls	German para-athletes vs. general population	Longitudinal, 8 time points	PHQ-4^a^, stress, training load	Para-athletes had lower PHQ-4 scores (p < 0.0001); weak training-stress correlation	Small sample, age mismatch, self-reported data
Lisa S. Olive et al. (2022)	427 athletes (71 para, 356 non-para)	Australian Institute of Sport-supported athletesa	Cross-sectional survey	GHQ-28^b^, K-10^c^, AUDIT-C^d^, BEDA-Q^e^	No significant differences in mental ill-health rates between groups; para athletes faced greater risk from adverse events, concussion, low psychological safety	Small para-athlete sample size, cross-sectional design, self-reported data
Joanna Sobiecka et al. (2019)	581 (470 athletes, 111 coaches)	Polish Paralympic athletes and coaches	Retrospective survey	Financial support, training facilities, coaching access	41.4% of athletes reported lack of regular financial support. 64.8% identified inadequate or no financial support. 20.4% noted limited access to specialized sports equipment. 13.0% highlighted lack of systematic medical care. 49.1% indicated absence of recruitment systems for disabled athletes. Statistically significant differences were found in funding, training access, and organizational support (p < 0.05).	Self-reported data, lack of pre-2010 baseline for comparison
Marte Bentzen et al.(2022)	13 Para athletes	Swedish elite Para athletes preparing for and participating in PG22	Longitudinal, mixed-method study over 22 weeks	PHQ-4 for anxiety and depression	Anxiety: Pre-PG22 (9.6% mild, 6.2% severe); During PG22 (9.5% mild, 38.1% severe); Post-PG22 (0% severe). Depression: Pre-PG22 (10.1% mild, 9% severe); During PG22 (19.1% mild, 19.1% severe); Post-PG22 (11.8% mild, 11.8% severe). Correlation r = 0.57, p < 0.001.	Small sample, no intellectual impairment data, limited generalizability, short follow-up.

^a^PHQ-4: Patient Health Questionnaire-4.

^b^GHQ-28: General Health Questionnaire-28.

^c^K-10: Kessler Psychological Distress Scale-10.

^d^AUDIT-C: Alcohol Use Disorders Identification Test-Consumption.

^e^BEDA-Q: Brief Eating Disorder in Athletes Questionnaire.

## Results

### Descriptive analysis

**Fig 1:**
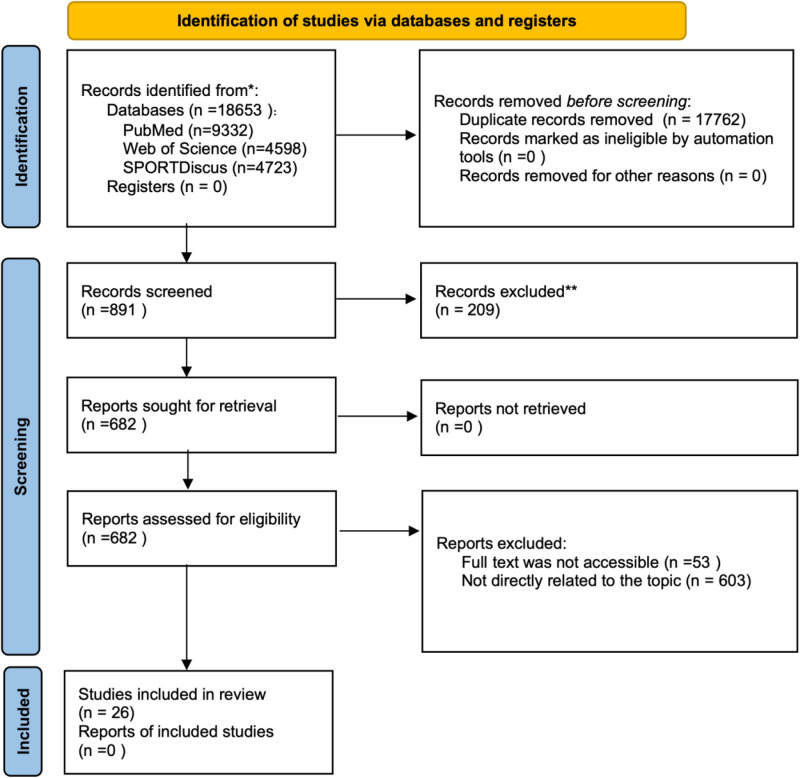
PRISMA flow diagram.

A total of 26 studies included in this review adhered to the inclusion criteria, focusing on societal attitudes and structural barriers in coaching athletes with disabilities. These peer-reviewed journal studies employed diverse methodological approaches, including quantitative descriptive studies, qualitative studies, and mixed-methods designs, allowing for a comprehensive exploration of the topic (Fig 1).

The geographical distribution of the studies contributed to a culturally diverse understanding of the issues. Studies were conducted in four major regions: Canada (n = 6), the United Kingdom (n = 3), Australia (n = 4), and multiple countries (n = 14), including six studies from low-income countries. This wide representation captured a range of cultural and environmental contexts, offering cross-national comparisons and highlighting both global challenges and region-specific nuances in coaching athletes with disabilities.

Methodological diversity further enriched the analysis. Among the included studies, 23 employed qualitative designs, 3 used quantitative descriptive methods, and 1 employed mixed-methods design. This range of methodologies enabled a comprehensive understanding of the complex intersection between societal attitudes and structural barriers in disability sports coaching.

The demographic characteristics of participants included both male and female athletes, ranging from adolescence to adulthood. Participants had various disability types, including physical disabilities (n = 12), intellectual disabilities (n = 3), and multiple disabilities (n = 12). Additionally, the athletes’ experience levels varied from novice to elite. While athletes with intellectual disabilities may face distinct challenges, those included in this study are preparing for the Paralympic Games, focusing on high-level competitive performance, like athletes with physical disabilities. This categorical representation ensured the review captured the distinct challenges associated with different impairment types and experience levels. This broad representation across demographics and skill levels is essential for understanding the complexities of coaching athletes with disabilities.

### Quality assessment

A total of 26 articles were assessed for quality using the MMAT, including 22 qualitative studies (Tables 4), 3 quantitative descriptive studies, and 1 mixed-methods review (Tables 5 and 6). Of these, 24 received the highest MMAT score, indicating high methodological quality, while 2 met 60% of the criteria. To ensure confidence in the synthesized findings, the GRADE-CERQual framework was applied, integrating MMAT results with assessments of coherence, adequacy of data, and relevance. Seven findings were rated with high confidence, while two received moderate confidence due to some data limitations. These results are summarized in the Summary of Qualitative Findings (SoQF) table (Table 7).

**Table 4:** Quality assessment results for qualitative studies.

Studies	Types	Evaluation Result							Overall MMAT Score
		S1^a^	S2^b^	1.1^c^	1.2^d^	1.3^e^	1.4^f^	1,5^g^	
William R. Falcão et al. (2015)	Qualitative review	Yes	Yes	Yes	Yes	Yes	Yes	Yes	5 (*****)
Y. Wareham et al. (2017)		Yes	Yes	Yes	Yes	Yes	Yes	Yes	5 (*****)
Y. Wareham et al.(2018)		Yes	Yes	Yes	Yes	Yes	Yes	Yes	5 (*****)
Janna L. Crawford and Monika Stodolska (2008)		Yes	Yes	Yes	Yes	Yes	Yes	Yes	5 (*****)
Lara Pomerleau-Fontaine (2021)		Yes	Yes	Yes	Yes	Yes	Yes	Yes	5 (*****)
Danielle Alexander, Gordon A. Bloom, and Shaunna Taylor (2019)		Yes	Yes	Yes	Yes	Yes	Yes	Yes	5 (*****)
Manuel Rodríguez Macías et al.(2023)		Yes	Yes	Yes	Yes	Yes	Yes	Yes	5 (*****)
Danielle Alexander et al.(2024)		Yes	Yes	Yes	Yes	Yes	Yes	Yes	5 (*****)
Andrea Bundonet al.(2022)		Yes	Yes	Yes	Yes	Yes	Yes	Yes	5 (*****)
Majed M. Alhumaid et al.(2022)		Yes	Yes	Yes	Yes	Yes	Yes	Yes	5 (*****)
Gallardo de León, Ponciano Nuñez, Calderón Santos (2024)		Yes	Yes	Yes	Yes	Yes	Yes	Yes	5 (*****)
Beth Aitchison et al.(2020)		Yes	Yes	Yes	Yes	Yes	Yes	Yes	5 (*****)
Olga Kolotouchkina et al.(2021)		Yes	Yes	Yes	Yes	Yes	Yes	Yes	5 (*****)
Damian Haslett et al.(2022)		Yes	Yes	Yes	Yes	Yes	Yes	Yes	5 (*****)
Emily Anne Rutland et al.(2022)		Yes	Yes	Yes	Yes	Yes	Yes	Yes	5 (*****)
Janine Coates & P. David Howe et al.(2023)		Yes	Yes	Yes	Yes	Yes	Yes	Yes	5 (*****)
Ian Brittain (2024)		Yes	Yes	Yes	Yes	Yes	Yes	Yes	5 (*****)
Yvette Wareham et al. (2018)		Yes	Yes	Yes	Yes	Yes	Yes	Yes	5 (*****)
Jacqueline Martins Patatas et al. (2020)		Yes	Yes	Yes	Yes	Yes	Yes	Yes	5 (*****)
Melinda A. Maikaet (2014)		Yes	Yes	Yes	Yes	Yes	Yes	Yes	5 (*****)
Darda J. Sales et al. (2022)		Yes	Yes	Yes	Yes	Yes	Yes	Yes	5 (*****)
Michelle Grenier et al. (2022)		Yes	Yes	Yes	Yes	Yes	Yes	Yes	5 (*****)

^a^Are there clear research questions?

^b^Do the collected data address the research questions

^c^Is the qualitative approach appropriate to answer the research question?

^d^Are the qualitative data collection methods adequate to address the research question?

^e^Are the findings adequately derived from the data?

^f^Is the interpretation of results sufficiently substantiated by data?

^g^Is there coherence between qualitative data sources, collection, analysis, and interpretation?

**Table 5:** Quality assessment results for quantitative descriptive studies.

Studies	Types	Evaluation Result							Overall MMAT Score
		S1^a^	S2^b^	1.1^c^	1.2^d^	1.3^e^	1.4^f^	1,5^g^	
Aglaja Busch et al.(2022)	Quantitative descriptive review	Yes	Yes	Yes	Partially	Yes	Partially	Yes	3 (***)
Lisa S. Olive et al. (2022)		Yes	Yes	Yes	Partially	Yes	Partially	Yes	3 (***)
Joanna Sobiecka et al. (2019)		Yes	Yes	Yes	Yes	Yes	Yes	Yes	5 (*****)

^a^Are there clear research questions?

^b^Do the collected data address the research questions?

^c^Is the sampling strategy relevant to address the quantitative research question?

^d^Is the sample representative of the target population?

^e^Are the measurements appropriate?

^f^Is the risk of nonresponse bias low?

^g^Is the statistical analysis appropriate to answer the research question?

**Table 6:** Quality assessment results for mixed methods studies.

Studies	Types	Evaluation Result							Overall MMAT Score
		S1^a^	S2^b^	1.1^c^	1.2^d^	1.3^e^	1.4^f^	1,5^g^	
Marte Bentzen et al..(2022)	Mixed Methods review	Yes	Yes	Yes	Yes	Yes	Yes	Yes	5 (*****)

^a^Are there clear research questions?

^b^Do the collected data address the research questions?

^c^Is there an adequate rationale for using a mixed methods design to address the research question?

^d^Are the different components of the review effectively integrated to answer the research questions?

^e^Are the results adequately brought together to answer the research questions?

^f^Are divergences and inconsistencies between quantitative and qualitative results adequately addressed?

^g^Do the different components of the review adhere to the quality criteria of each tradition of the methods involved?

**Table 7:** Summary of qualitative findings (SoQF) table.

Review Finding		Methodological Limitations^a^	Coherence^b^	Adequacy of Data^c^	Relevance^d^	Overall Confidence^e^
Societal Attitudes towards Athletes with Disabilities	Social Perceptions	Moderate	High	Moderate	High	Moderate
	Advocacy Through Communication	High	Moderate	High	High	High
	Representation Dynamics	High	Moderate	High	High	High
	Athlete Development and Equity	High	Moderate	High	High	High
Structural Barriers in Coaching	Environmental Barriers	High	Moderate	High	High	High
	Coaching and Development Dynamics	High	Moderate	High	High	High
	Structural Support Systems	High	Moderate	High	High	High
	Inclusive Environments	High	Moderate	High	High	High
	Psychological Resilience	Moderate	Moderate	Moderate	High	Moderate

^a^Assesses the reliability of the primary studies’ design and conduct contributing to the review finding.

^b^Evaluates how well the data from primary studies consistently support the review finding.

^c^Considers the richness and quantity of data underpinning the review finding.

^d^Examines the applicability of the evidence to the specific context of the review question.

^e^Combines the assessments to rate the confidence in the review finding as high, moderate, low, or very low.

**Fig 2:**
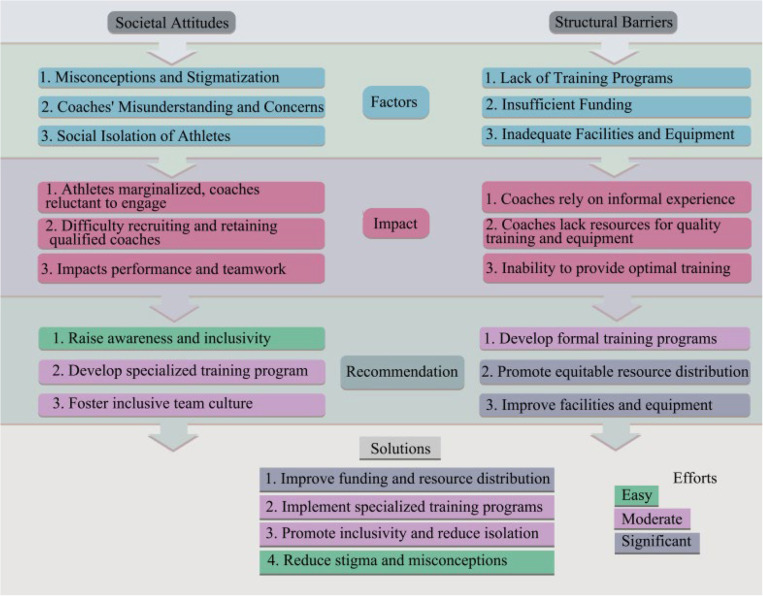
Framework for overcoming societal attitudes and structural barriers in coaching athletes with disabilities.

The findings from the included studies are grouped into a framework and presented in Fig 2.

### Social perceptions

Social perceptions surrounding disability are shaped by deeply ingrained societal narratives and systemic inequities that continue to marginalize individuals with disabilities, particularly in competitive and professional contexts [9,26]. Individuals with disabilities, particularly athletes, often encounter marginalization shaped by societal narratives that emphasize their impairments over their abilities [9,26]. The dominance of the medical model perpetuates a view of disability as a deficit requiring correction, framing individuals as dependent and less capable [26,27]. This perspective leads to systemic inequities, including underrepresentation in media, limited funding, and inadequate access to professional development and resources [26,27]. Athletes frequently report experiences of being treated as second-class competitors, with their sports often relegated to a symbolic or therapeutic role rather than recognized as legitimate and elite [28,29]. These stigmas not only undermine their self-esteem but also discourage broader participation in sports and societal integration.

In the Sport England (2001) survey, 19% of children with disabilities aged 6–16 reported that they did not participate in any sport due to societal inhibition or discrimination, highlighting the impact of these negative social perceptions. These perceptions not only shape participation in sports but also influence the broader societal integration of individuals with disabilities [30].

In many developing countries, these challenges are compounded by cultural and religious attitudes that frame disability as divine punishment or a curse, intensifying social exclusion and limiting opportunities for individuals to showcase their abilities [31]. In these contexts, negative societal attitudes towards disability persist, with individuals often treated as second-class citizens, undermining their opportunities for societal integration and participation in sports [32]. The perception of disability as something to be hidden or avoided creates additional layers of exclusion and marginalization [33].

Furthermore, media representation often reinforces these negative perceptions by framing athletes with disabilities as “superhuman” or “inspirational” due to their ability to overcome adversity, rather than focusing on their athletic prowess or competitive achievements [28,29]. This portrayal limits recognition of their professionalism, framing them as exceptions rather than skilled athletes [31,34]. Such narratives contribute to the marginalization of disability sports and undermine their legitimacy in broader societal contexts [31,34]. A 2012 study on media representation found that during the London 2012 Paralympic Games, 61.4% of articles framed disability in terms of athleticism, a positive portrayal, but 9.1% focused on the “medical/patient” aspect, and 9.1% used the “supercrip” narrative [35]. These portrayals limit the recognition of disabled athletes as legitimate competitors, reinforcing stereotypes and contributing to the marginalization of disability sports. Media coverage of disability sports remains disproportionately low, reinforcing stereotypes that these sports are less significant [31,36].

These entrenched social perceptions, which are particularly evident in developing countries, perpetuate the systemic barriers faced by athletes with disabilities [36,37]. Cultural resistance to inclusion, combined with the lack of support from governmental institutions, media, and society, makes it difficult to shift attitudes and provide equal opportunities for disabled athletes [35,38]. Addressing these issues requires a comprehensive approach to dismantling stereotypes, reshaping cultural attitudes, and fostering accurate, empowering narratives about disability sports [35,39]. True inclusion and equity for athletes with disabilities can only be achieved when these entrenched social perceptions are challenged and transformed [26,40].

### Advocacy through communication

Advocacy through communication serves as a powerful tool to reshape societal perceptions of disability, promoting inclusivity and enhancing the visibility of para-athletes [38]. In developing countries, communication plays an even more critical role in advocating for the rights and needs of athletes with disabilities [31–33]. Central to this approach is the development and implementation of innovative communication strategies [38]. Creative storytelling, such as anime-style content integrating Paralympic themes, has proven effective in engaging younger audiences and reshaping their understanding of disability [28]. Similarly, viral social media campaigns that use humor to address accessibility issues draw widespread attention to systemic barriers [28]. These strategies can be particularly impactful in developing countries where public awareness of disability sports is often limited [41]. Collaborations with major broadcasters, like NHK, and the inclusion of presenters with disabilities further strengthen efforts to balance empowering narratives while avoiding reductive stereotypes, paving the way for a more nuanced public discourse [28].

In the Polish study, 32.6% of athletes and 21.6% of coaches reported that a lack of understanding and acceptance of disabled athletes within sports associations is a significant barrier. Additionally, 17.4% of athletes and 21.6% of coaches noted difficulties in securing sponsorships, further underlining the importance of effective communication strategies in promoting disabled sports. Media coverage and communication strategies remain insufficient, with 27.9% of athletes and 19.8% of coaches pointing out that disability sports lack proper promotion. These findings suggest that while communication is crucial, there are still significant barriers in advocating for and promoting disabled athletes [30].

Citizen engagement plays an equally critical role in fostering support for para-sports and promoting early education on inclusivity [34]. In developing nations, community-based programs can help bridge gaps in societal attitudes towards disability [42]. Programs such as “Yoi Don!” and Paralympic-themed community events directly involve children, families, and local stakeholders, creating opportunities for meaningful interaction with para-sports [34]. However, the lack of infrastructure and cultural challenges in these regions require targeted advocacy efforts that engage local communities in disability-inclusive practices [39]. Structural challenges remain, including limited collaboration between schools and sports clubs, as well as insufficient outreach to parents and educators [34]. Addressing these gaps through targeted initiatives can bridge the divide, encouraging the participation of children with disabilities in sports and fostering a culture of inclusivity from a young age [30].

In developing countries like Kenya, Guatemala, and Ghana, the lack of media representation of para-athletes exacerbates societal stigma and stereotypes [31,32]. Media representation significantly influences how society perceives para-sports and individuals with disabilities [30]. Efforts to spotlight the personal achievements and stories of para-athletes in these regions can challenge negative societal attitudes, highlighting the resilience and talent of disabled individuals [33]. Highlighting the personal achievements and stories of para-athletes offers a powerful means to dismantle stereotypes and construct a more empowering narrative [30]. In many developing countries, amplifying the voices of athletes through local media can help change these narratives [43]. By normalizing disability within broader societal narratives and linking disability rights to other social movements, such as gender equality and LGBTQ+ advocacy, these efforts can attract wider public support and transform societal attitudes [43].

Ultimately, advocacy through communication demands an integrated approach that combines creative strategies, community engagement, and media reform [44]. By addressing systemic barriers and amplifying the voices of para-athletes, these efforts not only increase the visibility of para-sports but also contribute to broader societal change [44]. In developing countries, where challenges such as economic constraints, structural barriers, and societal stigma are more pronounced, advocacy through communication is a crucial tool to push for meaningful progress in the inclusion of athletes with disabilities [41]. The intersection of these elements forms a dynamic framework for promoting inclusivity, fostering understanding, and ensuring that the narratives surrounding disability reflect a more equitable and empowered reality.

### Representation dynamics

The representation of disability has long been shaped by the tension between the medical and social models. The medical model views disability as an individual deficit, often framed as an impairment needing correction or treatment [29]. Rooted in early rehabilitation efforts like the Stoke Mandeville Games, this perspective emphasizes overcoming physical limitations, reinforcing narratives of dependency and abnormality [29]. In contrast, the social model redefines disability as a societal construct, focusing on barriers created by inaccessible environments and exclusionary practices [32]. This approach advocates systemic change, challenging the notion that disability resides within the individual.

These perspectives are not entirely opposed, and integration is often necessary to address the multifaceted experiences of individuals with disabilities [32]. The social relational model acknowledges both physical impairments and the social stigma that amplifies exclusion. In para-sports, this synthesis is crucial, requiring both physical accommodation and societal reforms to foster inclusion. While impairments may impose certain limitations, societal perceptions and structural barriers often play a more significant role in marginalization [28]. In many developing countries, these structural barriers are intensified by economic hardship, cultural stigma, and a lack of resources, further marginalizing athletes with disabilities [31,39].

Stigma is a critical root cause that undermines representation and inclusion. Society often frames individuals with disabilities as either heroic exceptions or dependent victims, reinforcing harmful stereotypes [36]. In para-sports, this stigma reduces athletes to a “sub-elite” status, while coaches face fears of inadequacy or damage to their professional standing. Such attitudes, coupled with funding inequities and limited media coverage, further entrench exclusion and devalue para-sports [36]. In the London 2012 media study, 61.4% of articles framed disability in terms of athleticism, which is a positive portrayal, but 9.1% focused on the “medical/patient” aspect, and 9.1% used the “supercrip” narrative. These limited portrayals perpetuate stereotypes and hinder the recognition of disabled athletes as elite competitors, contributing to the marginalization of disability sports [35]. In developing countries, these stigmas are exacerbated by a lack of accessible sports infrastructure and financial constraints, making it harder for athletes with disabilities to compete on equal footing [42].

Efforts to challenge these stigmas are evident in programs like the Fakher Initiative and the Tokyo 2020 Paralympic Games [27]. These initiatives promote para-sports as elite competitions, emphasizing empowerment over deficit. Media innovations, including para-athletes’ involvement in storytelling and showcasing assistive technologies, aim to normalize disability and shift societal perceptions toward inclusion [27]. However, in developing nations, such initiatives are often hindered by limited media coverage and a lack of institutional support, making it crucial to increase visibility and funding in these regions [31,41].

An integrated approach is essential for lasting change. Frameworks like the ICF highlight the interplay between individual impairments and societal barriers [45]. Combating stigma requires more than policy reforms or infrastructure improvements; it demands a shift in societal attitudes [36]. By broadening media narratives, fostering inclusive education, and ensuring equitable access to resources, society can move beyond deficit-based perspectives and unlock the full potential of individuals with disabilities [35]. For lasting progress, it is vital to develop tailored strategies that address the specific barriers faced by athletes in developing countries, ensuring they receive the resources and recognition they need to thrive.

### Athlete development and equity

Athlete development in youth sports is hindered by systemic ableism, which marginalizes disabled athletes through exclusionary practices and structural inequities. Female athletes are particularly affected, often dismissed or infantilized by male coaches who lack the training to address their needs [46]. These issues are exacerbated by inadequate funding, inaccessible facilities, and limited adaptive equipment, which restrict opportunities for participation [46,47]. The scarcity of female coaches further compounds the problem, leaving disabled athletes without proper mentorship or advocacy within male-dominated systems [37]. Beyond gender, intersectional factors such as race and socioeconomic status further complicate access to equitable athlete development opportunities. Athletes from racial minority backgrounds often face additional discrimination, limiting their inclusion in sports programs that prioritize able-bodied and socioeconomically privileged participants [48]. In many cases, racial and ethnic biases influence funding allocation, with para-sports programs in marginalized communities receiving disproportionately fewer resources than those in wealthier or predominantly white areas [49].

In the Polish study, 26% of coaches reported that athletes faced challenges due to a lack of training resources and insufficient support for disabled athletes. Additionally, a comparison of para-athletes and non-para-athletes revealed that para-athletes were older on average (29.5 years) than non-para-athletes (22.7 years), which highlights the later entry into sports for many disabled athletes [30]. This delay in entry speaks to the barriers disabled athletes face in accessing sports from an early age. The study also found that 50.7% of para-athletes had completed tertiary education, compared to 29% of non-para-athletes, illustrating the additional challenges disabled athletes face in accessing educational opportunities [30].

In low-income countries, these challenges are often intensified by additional barriers such as societal discrimination, limited government support, and insufficient infrastructure, which hinder the development of para-athletes [32]. For instance, athletes from economically disadvantaged backgrounds often struggle to afford specialized training, adaptive equipment, or travel costs required for competitions, further limiting their participation in elite para-sports [32]. This financial burden disproportionately affects athletes from underprivileged racial and ethnic communities, who already face systemic barriers in accessing education, healthcare, and employment opportunities [33,50]. In resource-limited settings, para-athletes face unique challenges, including economic hardships, inaccessible facilities, ethnic favoritism, and a lack of professional coaching and resources [31,41]. Ethnic favoritism in sports administration can further exacerbate these disparities, with funding and selection processes often favoring athletes from dominant ethnic groups, sidelining minority para-athletes who already face multiple layers of exclusion [51]. However, initiatives such as the Fakher Disability Sports Programme in some of these regions show the positive impact of providing opportunities for para-athletes, fostering social inclusion and health benefits, despite the need for more psychological support, better facilities, and increased media coverage [42].

Despite these challenges, parasport offers profound social and psychological benefits, empowering athletes through enhanced confidence, independence, and resilience [52]. Participation fosters a sense of belonging by creating supportive communities where shared experiences reduce isolation and build self-esteem [30]. For athletes in resource-limited settings, the sense of community and inclusion in parasport is even more crucial, as it helps overcome societal stigma and marginalization [39]. However, intersectional barriers persist even within parasport communities. Athletes who belong to both disabled and marginalized racial groups often report feeling isolated, underrepresented in leadership roles, and excluded from major sponsorship and media coverage [28]. This lack of representation reinforces structural inequities, making it difficult for athletes from multiple marginalized identities to gain visibility and recognition [10,53].

Effective coaching plays a pivotal role, focusing on athletes’ abilities rather than limitations and fostering environments where both personal growth and athletic success are prioritized [43]. However, in economically disadvantaged regions, the shortage of qualified coaches and resources further exacerbates inequities, highlighting the need for specialized disability-specific coaching and better access to training programs [41]. Coaches also play a crucial role in addressing intersectional disparities by recognizing and actively countering biases that may affect their coaching methods, athlete selection, and overall support for diverse athletes. Training programs that incorporate intersectional awareness can help ensure that para-athletes of all racial, gender, and socioeconomic backgrounds receive equitable opportunities for development [54].

Parasport also drives societal change by challenging stereotypes and advocating for equity. By showcasing the capabilities of disabled athletes, it reshapes perceptions and promotes inclusion [42]. However, true inclusivity requires a broader effort to recognize and dismantle the systemic barriers that disproportionately affect athletes at the intersection of disability, race, and socioeconomic status [34,55]. Policies aimed at increasing funding for underrepresented communities, diversifying coaching staff, and ensuring equal representation in international para-sport events are critical steps toward achieving this goal [41]. Greater representation of disabled athletes from economically challenged areas, along with increased investment in adaptive sports programs, can help reshape societal attitudes and create more inclusive environments for para-athletes [39,56]. Greater representation of disabled athletes and leaders strengthens advocacy efforts and pushes for systemic reforms in funding, training, and access [44]. Through these efforts, parasport not only transforms individual lives but also contributes to a cultural shift toward greater acceptance and equity in sports and society.

### Environmental barriers

Environmental barriers in para-sports pose significant challenges, particularly in transportation, financial constraints, and resource inequality. Transportation systems often fail to accommodate athletes with disabilities, lacking features such as ramps or designated spaces, while discriminatory attitudes from operators worsen the issue [31]. For instance, 36.36% of athletes reported that discrimination and a lack of societal knowledge about disability hindered their sports participation [42]. Urban infrastructure, including uneven sidewalks and inaccessible sports facilities, further limits mobility and independence, making it difficult for athletes to access training and competition venues [31]. 45.45% of athletes mentioned the inaccessibility of sports facilities, such as narrow hallways and inadequate bathrooms, as significant barriers [10]. In low-income nations, these barriers are particularly acute due to inadequate infrastructure and resources. Many sports facilities are not accessible to disabled athletes because of poorly designed entrances, such as steps, and gym equipment that is not tailored to specific disabilities [31,33]. Emerging technologies, such as smart adaptive equipment and digital mapping tools, have the potential to mitigate these barriers. For instance, smart wheelchairs with automated terrain adjustments can improve mobility on uneven surfaces, while digital mapping applications designed for accessibility can help athletes identify barrier-free routes to training and competition venues, reducing transportation-related challenges [57,58].

Financial and logistical barriers are equally daunting. 53.57% of athletes reported significant economic barriers, with financial difficulties affecting their ability to train and compete [33]. The high costs of transportation, training, and specialized equipment force many athletes to prioritize basic needs over sports [33,46]. In undeveloped regions, economic constraints also play a critical role, where athletes often have to choose between food, shelter, and their sports career, leading to inadequate nutrition and limited resources for training [41,42]. Unpaid volunteer coaches and gender-based financial inequities exacerbate these challenges [33,46]. Additionally, long-distance travel to training facilities adds logistical stress and limits consistent participation, highlighting a systemic lack of investment in para-sports infrastructure and resources [41,59]. In low-resource areas, public transportation systems often fail to provide wheelchair access, further isolating athletes from opportunities to train [31,39]. 17.86% of athletes also pointed out that the low media visibility of Paralympic sports, especially women’s Paralympic sports, was a significant barrier to their success and recognition [60]. Virtual coaching technologies can serve as a critical solution by allowing athletes to train remotely with expert guidance, reducing their dependence on physical travel [61]. Online coaching platforms provide structured training programs tailored to athletes’ needs, enabling them to maintain consistent training regimens even in areas with inadequate sports infrastructure [62]. Additionally, motion-tracking software and wearable technology can be integrated into virtual coaching sessions to monitor athletic performance in real time, ensuring that athletes receive personalized feedback without requiring access to high-end training facilities [63,64].

Resource inequality compounds these issues, with limited access to specialized sports equipment, inadequate facilities, and insufficient professional support [52]. In developing countries, athletes often train in environments that are not designed to meet their specific needs, such as overcrowded and poorly maintained spaces, which can significantly hinder their progress [33]. Many para-athletes train in spaces not designed for their needs, often overcrowded and poorly maintained [52]. Stigma and neglect further reduce funding and opportunities, while a lack of tailored medical, nutritional, and coaching resources undermines their performance and development [30,43]. Societal attitudes toward disability also contribute to an environment where athletes are often marginalized and undervalued, with limited governmental support to protect their rights. The integration of adaptive sports equipment, such as exoskeletons and prosthetics with real-time biomechanical feedback, can help level the playing field by enhancing athletes’ physical capabilities in training environments with limited accessibility [65]. Additionally, advancements in 3D printing technology have made it possible to produce cost-effective, customized adaptive equipment, making specialized sports gear more affordable and accessible for para-athletes in resource-limited settings [66].

### Coaching and development dynamics

Tailored coaching and training play a crucial role in enhancing both athletic performance and personal development, especially for athletes in parasports [46]. Coaches must adapt their strategies to address individual needs, considering the physical, psychological, and emotional challenges faced by these athletes [46]. Effective approaches, such as autonomy-supportive practices and personalized problem-solving, foster confidence, independence, and intrinsic motivation [46,67]. Additionally, emotional support during critical transitions, such as entering parasports after acquiring impairments, strengthens athletes’ sense of belonging and reduces stress [37]. Collaboration with multidisciplinary support teams, including physiotherapists and psychologists, further enriches the training process by aligning technical preparation with mental resilience [37,51].

However, significant gaps exist in the availability of disability-specific content within formal coaching education. Current training programs rarely provide comprehensive guidance on supporting parasport athletes, leaving many coaches to rely on informal methods, such as mentorship or trial-and-error learning [54]. The lack of structured resources not only limits the development of adaptive techniques but also hampers the ability to address the diverse physical and emotional needs of athletes with disabilities [26,43]. Emerging technologies, such as adaptive equipment and virtual coaching, have the potential to fill these gaps by providing more flexible, accessible, and personalized solutions for both coaches and athletes [32,68]. For instance, virtual coaching platforms can offer remote coaching sessions, enabling coaches to reach athletes in underserved regions where in-person training is not feasible [69]. This technology also allows for more personalized feedback and continuous engagement, which is especially valuable in regions with limited resources [70]. Adaptive equipment, such as customized sports wheelchairs or assistive devices, further ensures that athletes with disabilities can train in environments tailored to their specific needs, overcoming infrastructure barriers [71].

In a survey, 100% of athletes emphasized the importance of their coaches for training and success. However, 64.80% of athletes reported changing clubs multiple times during their careers, which influenced their training experiences. Coaches also indicated that 80% valued peer relationships and mentoring as central to their approach. Yet, 63.64% of participants reported a lack of qualified coaches, leading to irregular and unstructured training. Additionally, during the pandemic, the subjective training load ranged from 491 minutes per week (T1) to 703 minutes per week (T8), highlighting variations in training intensity. Coaches emphasized the importance of individualized coaching to avoid conflicts and cater to unique needs [32].

In low-income countries, these gaps are further amplified by societal attitudes, limited access to qualified coaching, and inadequate infrastructure, which impede the development of tailored training programs [31–33]. Barriers like high costs, logistical challenges, and persistent stigma further exacerbate these shortcomings, diminishing the focus on tailored training [44]. In these contexts, athletes with disabilities often face additional economic and societal obstacles that make it difficult to access specialized coaching or resources, further limiting their opportunities for progression in parasports [41,42]. The integration of emerging technologies can play a pivotal role in overcoming some of these obstacles [71]. Virtual coaching platforms can reduce geographical and financial barriers, while adaptive equipment can be shared or rented in communities with limited access to personalized sports gear [72]. These technologies also help bridge the gap between limited local infrastructure and the need for specialized equipment and training environments.

To bridge these gaps, the development of specialized educational resources and certifications is essential. These should include modules on impairment-specific adaptations, communication strategies, and psychological support [46]. Moreover, training programs should incorporate the use of emerging technologies, providing coaches with the knowledge and tools to effectively integrate virtual coaching and adaptive equipment into their practices [69]. In low-income countries, where coaching resources and training programs are limited, there is a critical need for localized adaptations to coaching education, ensuring that coaches in these regions have access to specialized content that addresses the unique challenges athletes face [39]. Collaborative efforts between sports organizations, researchers, and coaches could also enhance resource accessibility and improve the quality of training [37]. Addressing these deficiencies would empower coaches in underdeveloped regions to provide more effective and inclusive guidance, ultimately fostering the athletic and personal success of parasport athletes. Additionally, only 12% of training programs for coaches currently include disability-specific content, indicating a clear need for more specialized education [44].

### Structural support systems

Structural support systems for para-athletes require a comprehensive approach that addresses both advocacy for greater recognition and integrated training strategies. Para-athletes face significant structural barriers, including financial constraints, inadequate media visibility, and limited societal recognition [73]. In low-income countries, the lack of funding for sports infrastructure, specialized equipment, and professional coaching severely hampers the development of athletes with disabilities [31]. Insufficient funding compared to Olympic athletes hampers their training and competition opportunities [73]. Furthermore, societal stigma and a lack of understanding about disabilities exacerbate these challenges, particularly in underdeveloped nations, where disability is often viewed through negative cultural lenses, further marginalizing these athletes and limiting their participation opportunities [32,33]. Addressing these issues necessitates a focus on equitable financial policies, systematic marketing efforts, and increased visibility in media to inspire participation and shift societal perceptions [33]. Advocacy through communication in these countries is essential, as it can help raise awareness of the challenges faced by para-athletes, including overcoming societal prejudices and securing the resources and respect they deserve [33]. Social recognition, supported by tangible and emotional resources, plays a critical role in sustaining athlete motivation and fostering greater integration of para-sports into broader cultural values.

Equally essential is the development of integrated training approaches that ensure holistic athlete development. Effective training frameworks combine psychological, technical, and physical fitness components, enabling athletes to achieve peak performance while maintaining balance [51]. Early technical and tactical preparation, supported by multidisciplinary teams of professionals such as psychologists, physiologists, and nutritionists, is critical for long-term success [51]. However, the accessibility of specialized equipment and facilities remains a persistent challenge, especially in low-income countries where the availability of adaptive infrastructure is often severely limited, underscoring the need for increased investment in such resources [41,52]. Collaborative learning methods, including mentorship and experiential training, enhance coaching effectiveness and allow for customized approaches tailored to athletes’ needs [30]. Programs integrating both able-bodied and disability-specific techniques not only facilitate knowledge sharing but also bridge gaps in training quality [30].

Policy and institutional support play a crucial role in shaping coaching practices for para-athletes. The lack of formalized policies regarding disability-inclusive coaching education creates inconsistencies in training quality and leaves many coaches unprepared to address the specific needs of para-athletes [51]. National and international sports organizations must implement standardized policies that require all coaching certification programs to include disability-specific training, ensuring that coaches are well-equipped to provide adaptive training strategies [67]. Additionally, sports governing bodies should mandate ongoing professional development programs that incorporate the latest advancements in adaptive sports technology, enabling coaches to integrate innovative techniques such as virtual coaching and biomechanical feedback into their training methods [37].

Furthermore, funding policies significantly impact the ability of coaches to develop effective training programs for para-athletes. Governments and sports federations must allocate targeted financial resources to support the recruitment, education, and retention of coaches specializing in disability sports [74]. In many low-income regions, financial constraints prevent coaches from accessing specialized training courses or participating in international knowledge-sharing initiatives [31]. Policy interventions should include subsidies or scholarships for coaches pursuing disability-specific certifications, along with financial incentives for those working in under-resourced areas [38]. These measures can help bridge the gap in coaching expertise and ensure that para-athletes receive high-quality training regardless of their geographic location. In a survey, 14.29% of athletes worked with physical trainers, while 85.71% relied on coaches for fitness training. Rehabilitation services, including physical therapy and sports-specific rehab, were offered in some programs, helping athletes recover and improve their performance [60]. However, 45.45% of athletes reported difficulty accessing sports facilities due to poor infrastructure. These accessibility issues are compounded by institutional constraints such as limited financial resources and societal attitudes towards disability, which remain systemic barriers to para-athlete development. In some programs, athletes received monthly payments of approximately 800 USD to support their training and living expenses, but the financial burden on athletes is still significant [33].

Addressing these interconnected challenges requires systemic reform at both societal and organizational levels. Advocacy efforts must be complemented by structured training programs that leverage interdisciplinary expertise to optimize support systems [43]. By integrating policy-driven strategies with grassroots coaching initiatives, national sports institutions can create a more sustainable coaching ecosystem that prioritizes inclusion and professional development. In developing countries, advocacy must go hand-in-hand with the creation of structured training programs that utilize interdisciplinary expertise to optimize support systems for para-athletes [39,42]. By enhancing financial, social, and infrastructural support while adopting holistic training methodologies, structural support systems can empower para-athletes to excel and achieve broader societal recognition.

### Inclusive environments

Inclusive environments, particularly within the domain of sports, serve as transformative spaces that foster empowerment and community belonging for individuals with disabilities. Empowerment through sports emerges as a pivotal mechanism by which participants develop critical skills, gain physical and psychological strength, and cultivate a sense of independence [52]. Tailored pathways and coaching strategies address the unique challenges faced by para-athletes, ensuring that development frameworks, such as the Long-Term Athlete Development (LTAD) model, are inclusive and adaptive [43]. These frameworks not only support physical literacy and technical proficiency but also enhance self-worth and provide opportunities for long-term growth.

Simultaneously, community and belonging are integral components of inclusive environments. Parasports create opportunities for individuals to build social networks, forming meaningful relationships with peers who share similar challenges and experiences [42]. This sense of belonging extends beyond athletes to their families, fostering a community where acceptance and shared purpose thrive. Parental involvement is highlighted as a critical element, offering emotional and logistical support and enhancing the athlete’s journey through collaboration with organizations and stakeholders [44]. In fact, 100% of athletes emphasized the importance of family support for success in sports. Social support also plays a crucial role, with 64.28% of athletes finding support from friends and teammates crucial, and all athletes agreeing on the importance of balancing friendships with athletic careers [60]. Inclusive coaching and structured programs further empower participants to construct positive identities and overcome societal and structural barriers, establishing environments that celebrate ability and diversity [39].

In developing countries, the creation of inclusive environments for athletes with disabilities faces significant challenges that exacerbate existing barriers [31,33]. Economic constraints force para-athletes to choose between training and basic survival, often leaving them without adequate resources to perform at their best [41]. Infrastructure and accessibility issues, such as the lack of ramps and accessible equipment, poor-quality facilities, and inaccessible public transportation, add logistical and financial burdens to their training [42]. 45.45% of athletes reported difficulty accessing sports facilities due to poor infrastructure [10]. Negative societal attitudes and cultural beliefs further contribute to exclusion and discrimination, limiting opportunities for these athletes [39]. Despite these obstacles, efforts to foster inclusive environments are crucial for the development and success of para-athletes [31]. In a survey, 72.73% athletes stressed the importance of education and awareness in improving social inclusion for people with disabilities, indicating a need for greater societal understanding [10]. Programs like Saudi Arabia’s Fakher Disability Sports Programme have shown progress through government support, access to competitions, and positive health outcomes, while also raising public awareness and building community among stakeholders [33]. However, there remains a need for more psychological support, better facilities, and increased media coverage to further promote disability sports. In Guatemala, para-athletes in Quetzaltenango face similar challenges, including economic barriers, social discrimination, and accessibility issues, highlighting the need for improved infrastructure, increased social awareness, and professional coaching support to create more inclusive sports environments [41]. Furthermore, 75% of athletes worked on physical fitness in an integrated way with technical and tactical training, underscoring the value of holistic approaches [38]. Government policies for integration often resulted in separate training programs for Paralympic athletes, limiting inclusiveness.

Overall, while progress has been made in some areas, there is a clear need for continued efforts to address resource constraints, improve accessibility, and challenge negative societal attitudes in developing countries. By doing so, stakeholders can better support the development of para-athletes and promote greater participation and success in sports.

### Psychological resilience

Psychological resilience, particularly within the context of Paralympic athletes, encompasses key elements such as emotional and psychological support, reliance on support networks, and the maintenance of psychological safety. These factors collectively contribute to individuals’ ability to adapt and thrive amidst challenges, including systemic inequalities and high-pressure environments [73].

Emotional and psychological support is fundamental to building resilience. Paralympic athletes have highlighted the importance of structured psychological interventions, which include working with sports psychologists to manage emotions, increase motivation, build confidence, and alleviate stress [73]. In fact, 100% of athletes stated that motivation, self-confidence, and managing pressure were critical to performance [33]. These strategies have been particularly vital during disruptions like the COVID-19 pandemic, as athletes relied on coping mechanisms derived from past trauma or life-altering injuries to maintain stability [75]. In the survey, 53.57% of athletes worked with sports psychologists, while 46.43% felt pressure from coaches [60]. Collaboration between athletes, psychologists, and coaching staff during high-stress events has also been critical in monitoring and reducing mental distress, thereby enhancing performance and wellbeing [10].

The dependency on support networks underscores the significance of interconnected relationships in fostering resilience. Athletes benefit from the emotional and logistical backing provided by families, friends, coaches, and medical teams. Integrated support systems, such as those offered through virtual platforms or financial sponsorships, further stabilize their mental health, compensating for systemic gaps in resources and equity [27]. 60.71% of athletes felt nervous and struggled to control their emotions before and during competitions, highlighting the emotional challenges they face in high-pressure situations [33]. Conversely, the absence of robust networks has been identified as a risk factor, highlighting the importance of cultivating accessible and equitable support environments [27].

In low-income countries, psychological resilience among elite athletes with disabilities is crucial for overcoming significant societal challenges [31]. These athletes face severe social stigma, being treated as “second-class citizens” and even experiencing discrimination [31]. Despite these adversities, they persist in their athletic pursuits and represent their countries on international stages, demonstrating remarkable psychological resilience [39]. Additionally, they contend with scarcity of resources, including lack of funding, inadequate training equipment, and inaccessible facilities. Yet, they remain committed to their sports, showcasing resilience in the face of such constraints [42]. Furthermore, these athletes maintain a positive outlook and a strong sense of self-efficacy [41]. In a survey, 54.55% of athletes expressed motivation to train despite economic and logistical barriers [10]. Their participation in sports not only helps them regain a sense of identity and manage the stigma associated with disability but also allows them to set goals and achieve a sense of accomplishment [41]. In a context where social support systems are weak and cultural beliefs often stigmatize disability, these athletes’ ability to persist and pursue their goals independently is a testament to their psychological resilience.

Psychological safety, as a protective factor, enables athletes to navigate the pressures of competition and societal expectations. Stress levels ranged from 2.8 ± 2.3 at T1 to 3.9 ± 2.4 at T8, with a weak positive correlation to training load, indicating that stress management remains a significant aspect of athletes’ mental well-being [76]. The assurance of immediate access to mental health resources fosters a secure environment where individuals feel comfortable addressing mental health concerns without fear of stigma or consequences [10]. Organizations promoting psychological safety through structured feedback and consistent support mechanisms have demonstrated positive impacts on athletes’ mental health outcomes, reinforcing the necessity of trust and inclusivity in competitive frameworks [10].

Ultimately, resilience is a dynamic interplay of internal and external mechanisms, shaped by prior experiences, adaptive strategies, and systemic supports [54]. The ability of para-athletes to reframe challenges positively and adapt to shifting goals reflects a profound psychological flexibility [77]. Programs tailored to enhance coping mechanisms, address impairment-specific stressors, and expand support networks are imperative for advancing resilience in this unique population. Paralympic athletes also demonstrated significantly better mental health, with PHQ-4 scores of 1.2 compared to 3.7 in the general population, indicating the psychological benefits of their engagement in sports [10].

### Impact on coaching practice

The coaching of athletes with disabilities is significantly shaped by societal attitudes and structural barriers, which present multifaceted challenges to practical coaching applications [34]. Extensive evidence from case studies and observational data underscores the pervasive influence of societal misconceptions about disability, which fosters an environment where athletes with disabilities are often marginalized and undervalued [41]. These attitudes are frequently framed by the medical model of disability, which views impairment as a deficit to be “fixed” rather than a difference to be accommodated. This approach not only stigmatizes athletes with disabilities but also discourages many coaches from engaging in parasport coaching. Specifically, coaches often express concerns about their ability to prevent injuries or achieve competitive success when working with disabled athletes, leading to a reluctance to specialize in this area [52]. In lower-income countries, these misconceptions are often exacerbated by deeply entrenched cultural beliefs that associate disability with misfortune or limitations, further reducing opportunities for effective coaching interventions. As a result, para-athletes in these contexts often receive suboptimal training or are excluded from mainstream sporting initiatives altogether [33,39]. Such societal attitudes compound the recruitment and retention difficulties for qualified coaches, creating a substantial gap in the availability of skilled professionals for athletes with disabilities.

In addition to societal attitudes, structural barriers such as limited access to specialized training programs, insufficient funding, and a lack of inclusive policies further exacerbate these challenges. Many coaches report that they lack formal education or professional development specific to coaching athletes with disabilities, forcing them to rely heavily on experiential learning or informal peer support [36]. This lack of preparedness results in suboptimal training environments where the diverse physical and psychological needs of athletes with disabilities are inadequately addressed [30]. The situation is particularly dire in less developed regions, where formal coaching education is often unavailable or inaccessible. Limited financial incentives further deter individuals from pursuing careers in para-sports coaching, leaving many athletes without skilled mentors to guide their development [42]. Moreover, the inequitable allocation of resources between parasport and able-bodied sport further hampers effective coaching [77]. Coaches working with athletes with disabilities frequently face challenges such as inadequate training facilities, insufficient access to specialized equipment, and limited financial support, all of which undermine their ability to provide high-quality coaching [30]. In many resource-constrained environments, para-athletes are forced to train with outdated or makeshift equipment, while coaches struggle to implement structured training programs due to a lack of basic infrastructure. For instance, many wheelchair basketball teams in low-income countries rely on secondhand wheelchairs, often ill-suited for competitive play. The absence of dedicated training centers also forces athletes to practice in unsuitable or unsafe conditions, further hindering their progress [41].

These structural barriers extend beyond the immediate coaching environment to broader institutional frameworks. Parasports often receive less media attention, public recognition, and financial investment compared to able-bodied sports, reflecting a broader societal undervaluation of disability sports [43]. For example, disparities in funding between parasport and able-bodied programs limit access to elite-level coaching, specialized training facilities, and essential sports equipment [42]. In lower-income countries, where overall sports funding is already scarce, para-sports receive only a fraction of the available resources, leading to chronic underdevelopment. Government policies and national sports federations often prioritize mainstream sports, leaving para-athletes reliant on sporadic donor funding or grassroots initiatives to sustain their training [33]. Without inclusive policies that mandate accessible environments and equitable resource distribution, athletes with disabilities are at a significant disadvantage.

Furthermore, the psychosocial dynamics within teams and the broader sporting community are heavily influenced by societal attitudes toward disability. Athletes with disabilities frequently face heightened psychosocial challenges, including isolation and exclusion, which can impair team cohesion and overall performance [35]. In these contexts, coaches play a critical role in fostering strong social relationships and promoting a positive and inclusive team culture [44]. However, the responsibility to overcome societal stigmas and create an inclusive environment often falls disproportionately on the coaches, requiring them to provide not only technical and physical training but also substantial emotional and psychological support [39]. This burden is particularly heavy in underdeveloped regions, where negative societal attitudes towards disability are compounded by economic hardship and limited community awareness. Female para-athletes often face additional obstacles due to gender biases, making it even more difficult to access coaching support and training opportunities. Coaches must navigate these intersecting challenges while advocating for greater inclusivity in sports [32].

In conclusion, the intersection of societal attitudes and structural barriers significantly impacts the practical realities of coaching athletes with disabilities. These challenges hinder the development of fully inclusive coaching environments, which are essential for the success of para-athletes. In lower-income regions, the situation is further exacerbated by a chronic lack of investment, cultural misconceptions, and systemic inequalities that disproportionately affect disabled athletes [31]. Addressing these issues requires comprehensive reforms, including the development of disability-specific coaching education programs, the implementation of inclusive sports policies, and equitable resource allocation. Governments, international sports organizations, and development agencies must prioritize investment in para-sports coaching, ensuring that both coaches and athletes have the resources they need to thrive [32]. To further illustrate the factors within societal attitudes and structural barriers, and our suggestions for removing these barriers, we propose a figure that outlines the necessary efforts (categorized as easy, moderate, or significant) to eliminate these obstacles (Fig 2). By tailoring these guidelines to specific settings and disabled sports, we can offer more practical and context-specific solutions. Only through systemic changes can athletes with disabilities gain access to the same high-quality coaching, facilities, and opportunities as their able-bodied counterparts. Without such reforms, the full potential of athletes with disabilities will remain unrealized, and the broader goal of inclusivity in sports will continue to be undermined.

## Discussion

This review reveals that societal attitudes and structural barriers are deeply intertwined, each reinforcing the other, with the challenges being particularly pronounced in developing countries. Negative societal perceptions of disability, often rooted in the medical model, contribute to systemic underinvestment in parasports. In developing countries, cultural attitudes that frame disability as a curse or divine punishment further marginalize individuals and limit opportunities for participation in sports. This lack of resources, whether in the form of accessible facilities, specialized coaching, or funding, creates additional obstacles for athletes with disabilities, perpetuating their marginalization. These barriers are not only physical but also influence the mindset of coaches, many of whom internalize societal biases and feel unprepared to work with para-athletes. This, in turn, limits the availability of high-quality coaching, further disadvantage athletes with disabilities. The intersection of these factors creates a cycle where societal and structural limitations reinforce each other, hindering progress in the field.

### Societal attitudes towards athletes with disabilities

This discussion critically examines the interplay of systemic, cultural, and individual factors influencing disability in sports, while integrating previous findings to highlight the value of this review’s contributions. A central debate emerges from the dichotomy between the medical and social models of disability, with significant implications for policy and practice [9]. The medical model, which frames disability as an individual deficit, has historically shaped societal attitudes but risks oversimplifying broader systemic issues, such as inadequate coach training and inconsistent policy implementation [38]. This perspective, while relevant, perpetuates dependency and marginalization by focusing on impairments rather than addressing structural barriers [36]. Conversely, the social model reframes disability as a societal construct, emphasizing systemic reforms to foster inclusion [26]. However, this model’s application remains inconsistent, constrained by entrenched medicalized attitudes. Hybrid frameworks like the ICF offer potential for reconciling these perspectives by integrating individual and structural considerations, though their implementation remains in early stages [26,78].

The representation of para-athletes in the media presents another critical tension. Dominant narratives often reduce para-athletes to “supercrips” or “passive victims,” celebrating individual achievements while neglecting systemic barriers [31]. While such portrayals amplify visibility and challenge biases, they risk undermining athletes’ recognition as competitive professionals by setting unrealistic expectations or diminishing agency [46]. Prior reviews, including this one, question the assumptions of progress in media representation, highlighting the persistence of reductive frames despite inclusive policies. Efforts like the Tokyo 2020 Paralympics, which adopted diverse storytelling strategies, demonstrate the potential for change but require broader cultural and structural shifts [28,79]. A balance between celebrating resilience and emphasizing athletic skill is essential for fostering genuine representation.

The therapeutic versus competitive framing of parasports further underscores contradictions in public and institutional perceptions. Therapeutic narratives emphasize personal and psychological benefits but risk relegating parasports to secondary status, limiting funding and professional recognition [80,81]. In contrast, advocates for competitive legitimacy argue for parity with able-bodied sports, emphasizing the need for equal resources and respect [38]. Integrating these perspectives through policies that acknowledge both therapeutic value and professional potential could enhance the holistic stature of parasports, aligning with findings that para-athletes face systemic exclusion despite growing opportunities [37].

Systemic inequities are also evident in resource allocation and classification systems, which disproportionately favor athletes with less severe impairments to maximize competitive success [30]. While proponents argue this enhances fairness, critics contend it marginalizes athletes with greater disabilities, exacerbating exclusion [82,83]. These findings challenge assumptions that existing frameworks are adequate, advocating for reforms to balance inclusivity with fairness to ensure equitable representation across impairment levels.

Coaching practices further highlight operational barriers to inclusion. Coaches often lack formal disability-specific training and default to specialized teams, citing perceived complexity in integrating athletes with disabilities [73]. Previous reviews identify this gap as a critical factor in disconnect between policy and practice, where para-athletes are seen as “exceptions” rather than integral competitors. Comprehensive training programs for coaches are necessary to bridge this divide and ensure equitable opportunities for development and competition [67,84].

Finally, systemic ableism in youth sports infrastructure exacerbates these challenges. Development frameworks, such as the Long-Term Athlete Development model, often fail to adapt to para-athletes’ unique needs, resulting in talent identification and retention gaps [27]. These findings underscore the compounded marginalization faced by para-athletes, particularly women, who experience intersecting barriers of ableism and sexism [77]. This intersectional perspective calls for further exploration of how gender, race, and class shape disability experiences in sports, a contribution that highlights this review’s unique value [32].

The challenges faced by athletes with disabilities are particularly pronounced in developing countries, where cultural, economic, and systemic barriers combine to create significant obstacles. Cultural attitudes that frame disability as a curse or divine punishment further marginalize individuals and limit opportunities for participation in sports [31]. These attitudes are compounded by limited media representation, which reinforces stereotypes and stigma [31,32]. In such contexts, para-athletes often face additional layers of exclusion due to economic constraints, lack of accessible infrastructure, and insufficient government support [32,41]. For example, in countries like Kenya, Guatemala, and Ghana, the lack of media coverage and resources hinders the development and visibility of para-sports [32,41].

However, innovative communication strategies and community engagement can play a crucial role in shifting these attitudes. Efforts such as creative storytelling, viral social media campaigns, and collaborations with major broadcasters have proven effective in engaging younger audiences and promoting inclusivity [32,41]. By amplifying the voices of para-athletes and highlighting their achievements, these initiatives can challenge negative societal attitudes and promote a more empowering narrative [42]. Additionally, linking disability rights to broader social movements, such as gender equality and LGBTQ+ advocacy, can attract wider public support and drive systemic change [42].

The representation and integration of para-athletes in sports require a comprehensive and multi-faceted approach. This discussion synthesizes previous findings with new insights, advocating for systemic reforms, inclusive media representation, equitable resource distribution, and improved coaching practices. The integration of hybrid frameworks like the ICF model and grassroots engagement initiatives is pivotal for dismantling systemic ableism and fostering sustainable change [33]. In the context of developing countries, targeted advocacy efforts and increased media representation are crucial to address cultural and economic barriers. By aligning individual, cultural, and institutional levels, these efforts can normalize para-sports and celebrate athletic excellence without compromising equity, advancing both scholarly understanding and practical inclusion in disability sports [43].

### Structural barriers in coaching

This review provides a significant contribution to parasports literature by examining the interconnected structural barriers facing both athletes and coaches. While previous studies emphasize para-athletes’ challenges, particularly in accessing facilities and adaptive equipment [85], this review broadens the scope by exploring systemic pressures on coaches. By addressing both perspectives, it highlights the compounded impact of these barriers on the sustainability of parasports [48].

Transportation and infrastructure remain critical challenges, with inaccessible public transport systems and facilities limiting participation. In low-income countries, these issues are particularly acute due to inadequate infrastructure and resources [31]. Critics of incremental improvements argue that these measures fail to address persistent inequalities, emphasizing the need for comprehensive urban planning reforms prioritizing universal design [86]. Financial constraints further exacerbate these challenges, with athletes and coaches facing high costs for specialized equipment, transportation, and nutrition [51]. In undeveloped regions, economic constraints also play a critical role, where athletes often have to choose between food, shelter, and their sports career, leading to inadequate nutrition and limited resources for training [32,42]. This study extends prior research by linking funding disparities to broader systemic issues, demonstrating how financial limitations reduce training quality and threaten program sustainability [42].

Resource inequality is another key issue, with athletes often relying on costly imports for adaptive equipment and sharing overcrowded facilities with able-bodied athletes [35]. In developing countries, athletes often train in environments that are not designed to meet their specific needs, such as overcrowded and poorly maintained spaces, which can significantly hinder their progress [39]. Integration into existing systems is insufficient without addressing para-specific needs, such as disability-focused coaching and dedicated resources [28,39]. Similarly, existing coaching frameworks lack disability-specific content, forcing coaches to rely on informal learning and limiting professional development opportunities [87]. In low-income countries, these gaps are further amplified by societal attitudes, limited access to qualified coaching, and inadequate infrastructure, which impede the development of tailored training programs [31–33]. This systemic neglect hinders recruitment and retention, further undermining the effectiveness of para-sport programs.

Psychological resilience plays a crucial role in para-athlete performance, yet disparities in access to mental health resources persist. In low-income countries, psychological resilience among elite athletes with disabilities is crucial for overcoming significant societal challenges [31]. These athletes face severe social stigma, being treated as “second-class citizens” and even experiencing discrimination [31]. Despite these adversities, they persist in their athletic pursuits and represent their countries on international stages, demonstrating remarkable psychological resilience [31]. Structured interventions like mental health monitoring and support networks mitigate some challenges but are constrained by systemic inequities and societal stigma [74]. Psychological safety, where athletes and coaches feel free to address concerns without fear of stigma, remains compromised by internalized ableism and unequal treatment compared to able-bodied peers [10,44]. These findings highlight the interconnectedness of psychological, infrastructural, and social barriers, emphasizing the need for systemic change.

While parasports are empowering, fostering confidence and skill development, these benefits are limited by societal stigma and inadequate developmental frameworks [40]. In developing countries, the creation of inclusive environments for athletes with disabilities faces significant challenges that exacerbate existing barriers [31,33]. Economic constraints force para-athletes to choose between training and basic survival, often leaving them without adequate resources to perform at their best [39]. Negative societal attitudes and cultural beliefs further contribute to exclusion and discrimination, limiting opportunities for these athletes [42]. Inclusive communities provide social and psychological support, but systemic and attitudinal barriers restrict athletes’ and coaches’ full integration [54]. Advocates of integration into existing systems highlight normalization benefits, but critics argue that such models often neglect para-athletes’ unique needs [34]. This review underscores the importance of hybrid approaches that balance integration with tailored pathways to address para-specific challenges.

In conclusion, this review deepens the understanding of parasports’ systemic barriers, emphasizing the need for comprehensive reforms in infrastructure, funding, and education to address these issues. Key recommendations include universal design, equitable funding policies, para-sport-specific coaching certifications, and enhanced psychological support systems [34,52]. In low-income countries, there is a critical need for localized adaptations to coaching education, ensuring that coaches in these regions have access to specialized content that addresses the unique challenges athletes face [41]. By addressing these interconnected challenges, stakeholders can build a sustainable and inclusive ecosystem for parasports, ensuring athletes and coaches thrive both competitively and socially. This holistic perspective enriches existing literature while offering actionable strategies to advance equity and inclusion in parasports.

### Practical implications for coaches

The findings underscore the critical role of coaches in shaping the experiences and success of para-athletes, emphasizing the need for targeted interventions. Given the significant impact of societal attitudes and structural barriers on coaching practice, as highlighted in the results section, it is crucial to address these challenges comprehensively. This is especially true in lower-income regions where the challenges are compounded by cultural misconceptions and systemic inequalities.

First, coaching programs should integrate specialized training on disability inclusion and adaptive techniques, ensuring coaches are equipped to address the diverse needs of para-athletes [26]. In lower-income countries, where formal coaching education is often unavailable or inaccessible, efforts should be made to develop accessible and affordable training modules that can be delivered through online platforms or community-based workshops [31,32]. Without structured education, reliance on trial-and-error approaches may hinder athlete development and competitive potential [26]. Coaches must also develop strategies to navigate systemic barriers, such as limited access to adaptive equipment and accessible facilities [29]. By fostering problem-solving skills and collaboration with athletes, administrators, and stakeholders, coaches can create more inclusive training environments.

Additionally, coaches should be aware of intersectional challenges, particularly related to gender and other social identities, to prevent reinforcing exclusionary practices and promote equity [88]. In many developing regions, female para-athletes face additional obstacles due to gender biases, making it even more difficult to access coaching support and training opportunities [33]. Coaches must actively work to challenge these biases and create inclusive spaces for all para-athletes. The review also suggests that coaches can act as advocates for systemic change by engaging in initiatives that secure better resources and support for para-sports, thereby contributing to the sustainability of these programs. This is especially important in contexts where parasports receive limited funding and attention compared to able-bodied sports. By advocating for inclusive policies and equitable resource allocation, coaches can help bridge the gap between mainstream and para-sports.

### Policy and institutional recommendations

This review highlights the need for policy reforms and institutional support to address structural barriers faced by para-athletes and coaches. Sports administrators, policymakers, and advocacy groups play a crucial role in shaping inclusive sports environments by influencing policy development, resource allocation, and public awareness initiatives [31,49]. These stakeholders must work collaboratively to create and implement regulations that ensure the equitable treatment of para-athletes at all levels of competition. Sports organizations must prioritize the development and enforcement of policies that mandate accessible and inclusive training environments, ensuring all facilities are equipped with adaptive equipment and infrastructure that accommodate a range of disabilities [31]. Policies should also emphasize integrating disability-specific training into coach education programs, with national and international sports institutions collaborating with disability organizations to create standardized certification courses. In developing countries, where resources are limited, policies should also focus on the efficient allocation and utilization of available resources to maximize accessibility and inclusivity [31].

Policies should emphasize integrating disability-specific training into coach education programs, with national and international sports institutions collaborating with disability organizations to create standardized certification courses. In low-income regions, where formal coaching education is often unavailable or inaccessible, efforts should be made to develop accessible and affordable training modules that can be delivered through online platforms or community-based workshops, addressing the unique challenges faced by athletes in these areas [31,32]. Sports administrators must also be proactive in integrating disability-inclusive policies into national sports strategies [37]. This includes developing targeted funding mechanisms that prioritize accessibility, fostering partnerships between disability advocacy groups and mainstream sports organizations, and implementing regular assessments to measure the effectiveness of inclusion policies in para-sports programs [73].

Moreover, policy enforcement must bridge the gap between guidelines and practical application. Institutions should implement monitoring mechanisms to ensure inclusive practices are not tokenistic but embedded in daily operations [49]. Policymakers must establish regulatory frameworks that hold sports federations accountable for implementing accessibility standards, ensuring compliance through regular audits and performance evaluations. Additionally, advocacy groups play a vital role in amplifying the voices of para-athletes and pressuring institutions to uphold their commitments to inclusivity [67]. Their involvement in policy discussions and public campaigns can help challenge discriminatory practices and push for systemic changes. In developing countries, where cultural attitudes and economic constraints significantly impact para-sports, policies should also address these underlying issues by promoting awareness campaigns and community engagement initiatives to challenge negative societal attitudes and stereotypes [33].

Financial investment is crucial to overcoming systemic barriers. Policymakers should advocate increased funding dedicated to para-sports programs, supporting facility improvements, equipment procurement, and sustainable pathways for coach recruitment, training, and retention. Government agencies and sports administrators must collaborate to create dedicated funding streams for para-sports, ensuring that resources are allocated equitably between able-bodied and disabled athletes [60]. This financial support should extend to grassroots development programs, talent identification initiatives, and adaptive equipment subsidies to increase participation and competitive opportunities. In developing countries, targeted advocacy efforts and increased media representation are crucial to address cultural and economic barriers, attracting wider public support and driving systemic change [42]. Without adequate funding and support, para-sport programs risk stagnation, further marginalizing athletes with disabilities.

Lastly, institutional change should promote a broader cultural shift in how para-sports are perceived and supported. Organizations must champion narratives that position para-athletes as integral to the sports ecosystem rather than as exceptions [37]. Sports administrators and policymakers must actively work to integrate para-sports into mainstream sporting events and governing structures, ensuring that para-athletes receive the same level of recognition, sponsorship opportunities, and professional support as their able-bodied counterparts [32]. Advocacy groups should continue to push for inclusive media representation, increased broadcasting of para-sports competitions, and the inclusion of para-athletes in decision-making bodies to strengthen their influence on policy and program development [33]. This cultural transformation can be facilitated by creating platforms for para-athletes and coaches to voice their experiences and influence decision-making processes. In developing countries, linking disability rights to broader social movements, such as gender equality and LGBTQ+ advocacy, can attract wider public support and drive systemic change [32]. Through a combination of policy reform, institutional support, and cultural change, the structural barriers identified in this review can be addressed, promoting a more equitable and sustainable future for para-sports.

### Limitations and future research

This systematic review has several limitations. First, while previous studies predominantly focused on high-income countries, this review has incorporated research from developing countries, which provides insights into the unique challenges faced by athletes with disabilities in low-resource settings. Future research should continue to focus on diverse geographic and socio-economic contexts to further explore how different regions address societal attitudes, infrastructural barriers, and resource availability for athletes with disabilities. Additionally, while qualitative studies offer valuable insights, the predominance of such studies limits the ability to make broad generalizations. Future research should focus on more quantitative and longitudinal studies to empirically assess the long-term impacts of societal and structural barriers across various sports environments. Furthermore, the studies included in this systematic review often recruited both Paralympic and non-competitive athletes with disabilities as well as both physical and intellectual disabilities in one study, while majority of studies reported from a Paralympic perspective. These characteristics and types of disabilities may result in very different challenges and difficulties for the athletes with disabilities. Also, Para-athletes and recreational athletes with disabilities may face very different challenges in societal attitudes and structural barriers. Future research needs to investigate these issues. Due to the limitation of the included studies, this systematic review is not able to investigate these issues.

While emerging technologies such as adaptive equipment and virtual coaching are increasingly recognized, there is still limited research on how these innovations address infrastructural barriers and improve coaching practices. Future studies should explore the effectiveness of these technologies in overcoming geographical, financial, and infrastructure barriers, particularly in resource-limited settings.

Furthermore, the perspectives of sports administrators, policymakers, and advocacy groups were explored in this review. However, future research should further examine how these groups can collaborate more effectively to develop policies that directly address gaps in para-sport infrastructure, funding, and coach training.

Although gender-related challenges were discussed, the intersectionality of race, gender, and socio-economic status was appropriately addressed, but more work is needed to understand the specific ways these factors intersect in different cultural contexts. Future research should continue to explore how athletes with disabilities experience these compounded challenges across various identities, and how policies and practices can be better tailored to support them.

By addressing these limitations, future studies can provide a more nuanced understanding of the challenges and opportunities in coaching athletes with disabilities, ultimately supporting the development of more effective and inclusive strategies.

## Conclusions

This review highlights how societal attitudes, rooted in the medical model of disability, and structural barriers, such as inadequate resources and coach education, marginalize athletes with disabilities. Despite the existence of inclusive policies, gaps in implementation persist, and female para-athletes face additional challenges due to the intersection of ableism and sexism. Addressing these issues requires comprehensive reforms in coach training, resource allocation, and policy enforcement, alongside a cultural shift to view para-athletes as equal competitors. Only through such systemic changes can true inclusiveness in sports be achieved.

## Supporting information

S1 File PRISMA 2020 checklist. (DOCX)
